# Recent range shifts of moths, butterflies, and birds are driven by the breadth of their climatic niche

**DOI:** 10.1093/evlett/qrad004

**Published:** 2023-03-12

**Authors:** Maria H Hällfors, Risto K Heikkinen, Mikko Kuussaari, Aleksi Lehikoinen, Miska Luoto, Juha Pöyry, Raimo Virkkala, Marjo Saastamoinen, Heini Kujala

**Affiliations:** Research Centre for Environmental Change, Organismal and Evolutionary Biology Research Programme, Faculty of Biological and Environmental Sciences, University of Helsinki, Helsinki, Finland; Nature solutions unit, Finnish Environment Institute (Syke), Helsinki, Finland; Nature solutions unit, Finnish Environment Institute (Syke), Helsinki, Finland; Nature solutions unit, Finnish Environment Institute (Syke), Helsinki, Finland; Finnish Museum of Natural History, University of Helsinki, Helsinki, Finland; Department of Geosciences and Geography, Faculty of Science, University of Helsinki, Helsinki, Finland; Nature solutions unit, Finnish Environment Institute (Syke), Helsinki, Finland; Nature solutions unit, Finnish Environment Institute (Syke), Helsinki, Finland; Research Centre for Environmental Change, Organismal and Evolutionary Biology Research Programme, Faculty of Biological and Environmental Sciences, University of Helsinki, Helsinki, Finland; Helsinki Institute of Life Science, University of Helsinki, Helsinki, Finland; Finnish Museum of Natural History, University of Helsinki, Helsinki, Finland

**Keywords:** Aves, distribution, global change, Lepidoptera, pre-adaptation, phenotypic plasticity

## Abstract

Species are altering their ranges as a response to climate change, but the magnitude and direction of observed range shifts vary considerably among species. The ability to persist in current areas and colonize new areas plays a crucial role in determining which species will thrive and which decline as climate change progresses. Several studies have sought to identify characteristics, such as morphological and life-history traits, that could explain differences in the capability of species to shift their ranges together with a changing climate. These characteristics have explained variation in range shifts only sporadically, thus offering an uncertain tool for discerning responses among species. As long-term selection to past climates have shaped species’ tolerances, metrics describing species’ contemporary climatic niches may provide an alternative means for understanding responses to on-going climate change. Species that occur in a broader range of climatic conditions may hold greater tolerance to climatic variability and could therefore more readily maintain their historical ranges, while species with more narrow tolerances may only persist if they are able to shift in space to track their climatic niche. Here, we provide a first-filter test of the effect of climatic niche dimensions on shifts in the leading range edges in three relatively well-dispersing species groups. Based on the realized changes in the northern range edges of 383 moth, butterfly, and bird species across a boreal 1,100 km latitudinal gradient over c. 20 years, we show that while most morphological or life-history traits were not strongly connected with range shifts, moths and birds occupying a narrower thermal niche and butterflies occupying a broader moisture niche across their European distribution show stronger shifts towards the north. Our results indicate that the climatic niche may be important for predicting responses under climate change and as such warrants further investigation of potential mechanistic underpinnings.

## Introduction

The distribution of biodiversity around the globe is strongly affected by climatic conditions (Parmesan & Yohe, 2003; Pigot et al., 2010; Thuiller et al., 2008). The on-going changes in climate will evidently affect species ranges, as suitable conditions, e.g., suitable thermal niche space, shift towards the poles and higher altitudes. Research has shown that range shifting is a prominent and adaptive strategy for responding to both past and contemporary climate change (Davis & Shaw, 2001; Donoghue, 2008; MacLean & Beissinger, 2017; Parmesan et al., 1999). Many studies have identified both a general turnover in community composition (Devictor et al., 2012; Pecl et al., 2017; Pilotto et al., 2020; Richard et al., 2021) and increasing influx of warm-dwelling species along with climate change (Antão et al., 2020; Hickling et al., 2005; Virkkala & Rajasärkkä, 2011).

There is, however, large variation among species in how much the species de facto shift their distributions along with climate change (Champion et al., 2021; Parmesan & Yohe, 2003; Williams & Blois, 2018), and clearly all species do not respond to climate change through range shifts. Understanding which species can track shifting climatic conditions thus remains a central question in understanding species adaptation potential to climate change. This is crucial for both identifying species likely to suffer from climate change and to predict the redistribution of biodiversity and changes in species assemblages across space. To explain the variation among species, previous studies have assessed the role of external factors, such as differences in climate change velocities in inflicting differing magnitudes of environmental change on different species (Lenoir & Svenning, 2015; Pinsky et al., 2013; Williams & Blois, 2018), and on intrinsic factors, such as variation in species morphological and life-history traits, like generation time, size, dispersal ability, or habitat use, making species prone to differing responses to environmental change (Betzholtz et al., 2013; Brommer, 2008; Fourcade et al., 2021; Franzén et al., 2020; Pöyry et al., 2009). Despite the alleged logic that morphological and life-history traits would offer predictive potential for range shift responses, several meta-analyses have failed to identify a general connection between such traits and range shifts (Angert et al., 2020; Beissinger & Riddell, 2021; Buckley & Kingsolver, 2012; MacLean & Beissinger, 2017).

Recent studies have indicated that ecological attributes describing affinity to climatic conditions offer potential to understand the differences seen in species’ responses to environmental change via range shifts (Amano et al., 2014; Beissinger & Riddell, 2021; Day et al., 2018; Herrera et al., 2018; Scridel et al., 2017; Socolar et al., 2017; Thurman et al., 2020). The mechanistic underpinning is theorized to be formed by species’ composite tolerance towards abiotic conditions, as it functions as a means to persist in situ (Buckley & Kingsolver, 2012; Buckley & Puy, 2022; Matesanz et al., 2010; Nicotra et al., 2010). In essence, a species’ current climatic tolerance reflects how long-term adaptation and range shifts as a response to past climatic conditions have shaped the array of conditions effectively exploitable to the species (Lancaster, 2022). Such large-scaled patterns in niche breadth variation may affect how species respond to on-going changes (Hoffman et al., 2003). Some species are more specialized in regard to climatic conditions while others can tolerate a broader range of temperatures, precipitations, and/or seasonal conditions. According to van Valen’s niche variation hypothesis (Van Valen, 1965), populations that occupy a broader niche should be physiologically more tolerant compared to populations with narrower niches. Thus, we can hypothesize that a species occupying more variable climatic conditions across its range would be more climate resilient, and consequently would not predominantly need to respond through spatial shifts as the average conditions change. Instead, these species could stay and cope, or adjust plastically or evolutionarily (Beissinger & Riddell, 2021; Carscadden et al., 2020; Herrera et al., 2018; Reif & Flousek, 2012). In contrast, a more specialized species with an overall lower flexibility towards climatic variation may be relatively more inclined to efficiently track suitable conditions across space (Herrera et al., 2018; Hoffmann et al., 2003; Stefanescu et al., 2011). More efficient tracking of preferred environmental conditions may result from trade-offs between specialization and habitat-seeking ability during evolutionary history (Jacob et al., 2018). Species with narrower tolerances could also experience stronger selection pressure for dispersal at their leading range edge (Bridle et al., 2014; Saastamoinen et al., 2018; Travis et al., 2013) compared to species with broad tolerances (assuming that individuals at the leading edge share the same tolerance breadth as measured across the species’ distribution). Theory predicts and studies have shown that species inhabiting climatically more variable areas at higher latitudes may have broader tolerance to abiotic conditions (Rapoport’s rule; Deutsch et al., 2008; Stevens, 1989). Thus, species occupying relatively warmer conditions could be shifting their ranges more than those with cooler niches, as species distributed across lower latitudes would tend to also have narrower niches (the so called Rapoport’s rule; Stevens, 1989). This could explain recent findings of more southerly distributed species showing stronger spatial shifts with climate change. However, the explanatory role of niche breadth, which may correlate with mean niche value at least for temperature and thus offer a more proximate explanation for range shifts has rarely been tested under contemporary climate change conditions.

In this study we test the connection between observed range shifts and the mean and breadth of species’ climatic niche (cf. the Species Temperature Index [STI] concept; Devictor et al., 2012), as measured across the European distribution of 239 moth, 57 butterfly, and 87 bird species. Specifically, we quantify the shift in the northern facet of distributions in Finland, northern Europe, as shifts in the leading range edge are likely more directly driven by impacts of climatic conditions and thus more readily observed and measurable from distribution data than shifts in the trailing edge (Massimino et al., 2015; Pearce-Higgins & Green, 2014; Thomas et al., 2006). We also test for the effect of range size and morphological and life-history traits, which are commonly assumed to predict species abilities to track the changes in climatically suitable areas. Previous studies on these species groups in the boreal region have shown that species composition is changing (Antão et al., 2020; Virkkala & Lehikoinen, 2017) and that many species, but not all, are shifting their ranges northwards (Brommer, 2004; Brommer et al., 2012; Fourcade et al., 2021; Hällfors et al., 2021; Lehikoinen & Virkkala, 2016; Pöyry et al., 2009; Virkkala & Lehikoinen, 2014). In several of these studies the effect of traits has been tested, but this is the first study to relate range shifts to climatic niche metrics, which have shown the potential to help explain variation in direction and magnitude of shifts in studies from other parts of the world (Day et al., 2018; Herrera et al., 2018; Scridel et al., 2017). We hypothesize that species with relatively narrow niches would show stronger advancement of their distribution area towards the north compared to species with a broader niche.

## Methods

### Study species and area

Moths, butterflies, and particularly birds are relatively mobile species that have much potential to move across geographical space with climate change. Previous studies have, however, indicated large variation in range shift responses among these species (Brommer, 2004; Brommer et al., 2012; Fourcade et al., 2021; Hällfors et al., 2021; Lehikoinen & Virkkala, 2016; Pöyry et al., 2009; Virkkala & Lehikoinen, 2014). To help explain this variation, we make use of distribution data from across Europe to estimate the mean and breadth of the climatic niches of the species, and how such long-term evolutionary patterns in climatic adaptation may affect contemporary changes at the leading edge of the species’ ranges. Our study area, Finland, covers a latitudinal gradient of over 1,100 km. The climatic isoclines and forest vegetation zones within this boreal and subarctic bioclimatic region roughly follow latitudes (Ahti et al., 1968) and the northern range margins of many temperate and boreal species are situated in Finland (Pöyry et al., 2009; Virkkala & Lehikoinen, 2014). Thus, our study area is well suited for analyzing recent shifts in leading range edges and focuses on the period of a most prominent warming of 0.2–0.4 °C per decade (Mikkonen et al., 2015). Thus, our data can be expected to show relatively strong responses to climate change if species have indeed been able to respond through range shifts.

### Data

#### Distribution data

For moths and butterflies, we sourced observations available from the Insect database and National Butterfly Monitoring Scheme (NAFI; Saarinen et al., 2003), through the Finnish Biodiversity Information Facility (FinBIF; Supplementary Texts S1 and S2). The data were divided into two five-year periods: 1992–1996 (hereafter T_1_) and 2013–2017 (hereafter T_2_) and converted into presence-only data for each 10 × 10 km grid square. The total number of presence squares in T_2_ was substantially higher than in T_1_ due to increased sampling effort over time. To account for the change in sampling effort, we divided the data into five latitudinal zones (Supplementary Figure S1) and randomly subsampled the observations in T_2_ across the species so that they matched the number of observations in T_1_ within the latitudinal zone. This was repeated five times leaving us with five subsets of the data on Lepidoptera, where the total number of observations of all species combined was equal between the period for each latitudinal zone.

For birds, we used distribution data on terrestrial breeding birds, sourced from three national bird atlases through the Finnish Museum of Natural History (Supplementary Texts S1 and S2). These atlases have been compiled from national bird surveys carried out during 1974–1979, 1986–1989, and 2006–2010. As the third atlas has been surveyed more extensively in comparison to first two atlases, and following earlier practices, we used the pooled first and second atlas data, covering the period 1974–1989 (hereafter T_1_) and compared this to the third (2006–2010) atlas (hereafter T_2_) to reduce potential observation biases due to differences in survey effort (Kujala et al., 2013; Virkkala & Lehikoinen, 2017).

**Figure 1.**
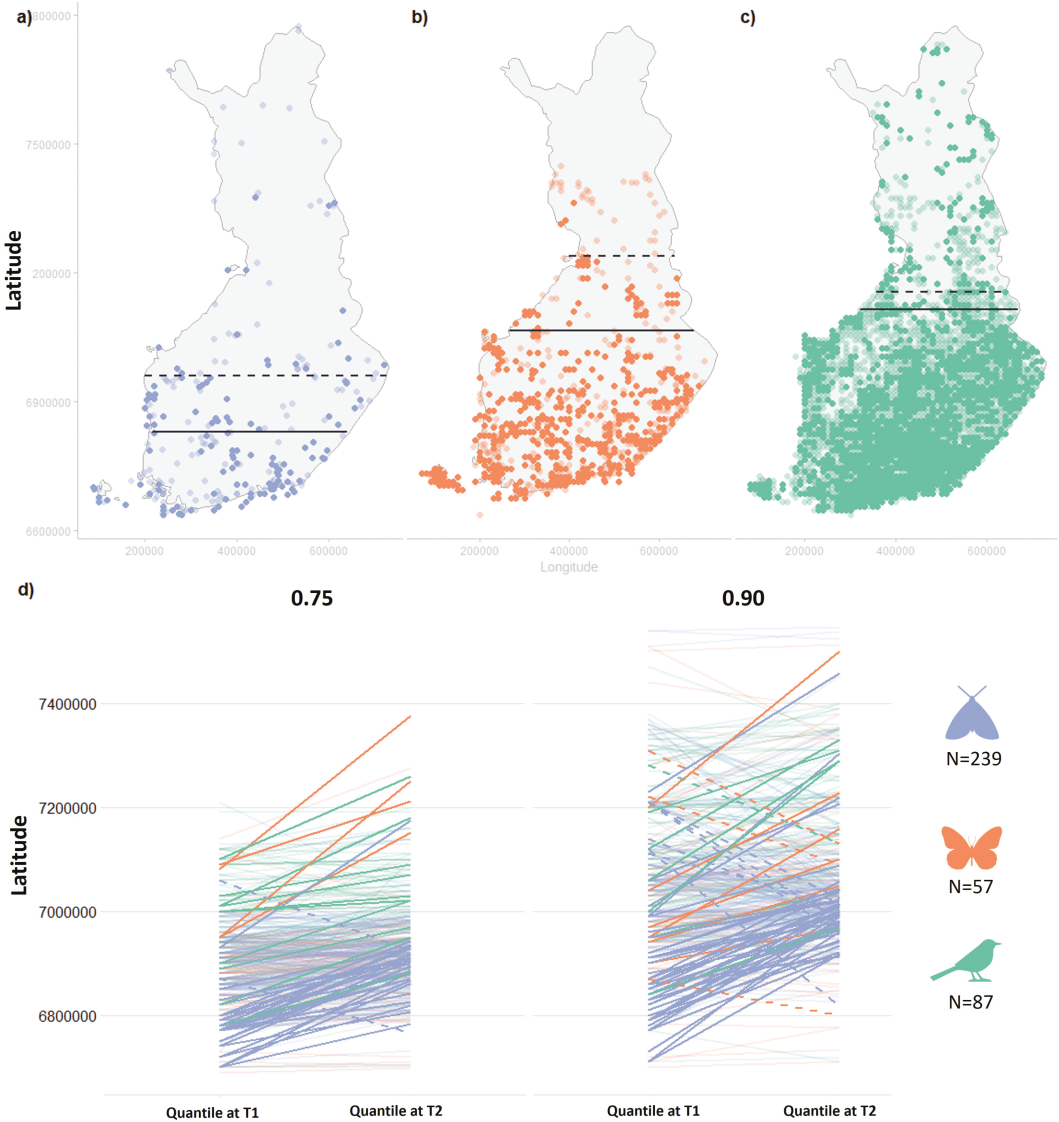
Range shifts were quantified as the shift in the 0.9 and 0.75 quantiles of distribution records using quantile regression. Examples of shift in the 0.9 quantile for (A) the moth species *Cerapteryx graminis,* (B) the butterfly species *Lycaena virgaureae*, and (C) the bird species *Troglodytes troglodytes*. Dark colored points and line represent distribution points and 0.9 quantile, respectively, at T_1_ and lighter points and dashed line represent distribution points and 0.9 quantile, respectively, at T_2_. For moths and butterflies the distribution points in T_2_ are from one of the five subsampled data sets. Panels (D) and (E) show shift in 0.75 and 0.9 quantiles of the distribution across a latitudinal gradient for all species (*N* = 383), colored by species groups. Faded lines = poor evidence for shifts, i.e., confidence interval (CI) for the estimated difference in quantile between T_1_ and T_2_ includes zero; full colored lines = CI for a positive (solid lines) or negative (dashed lines) estimated difference in quantile does not include zero. Latitudes and longitudes in projection format ETRS-TM35FIN, EPSG:3067. See Supplementary Figures S4 and S5 for corresponding plots to (D) but divided per response type (northward, southward, and no shift), and per taxonomic group and response type, respectively. Moth icon by Miroslava, butterfly icon by Arthur Shlain, bird icon by Hea Poh Lin, all via the Noun Project https://thenounproject.com.

We included only species that likely have their northern range borders in Finland by removing species for which the center point of distribution in Finland in T1 was ≥ 7,000,000 north in the Finnish uniform coordination system (63°4ʹN in degrees; range in Finland 59°46ʹ–70°5ʹN; Supplementary Figure S1) (Brommer, 2004; Brommer et al., 2012; Kujala et al., 2013). This allowed us to focus on the predominantly southern species with leading distribution edges in Finland. After data delimitation (Supplementary Text S1) the data available for the main analyses represented 383 species: 239 species of moths, 57 species of butterflies, and 87 species of birds for which the shift in the northern facets of distribution between two periods of time, roughly 20 years apart, were measured (example the data for three species in Figure 1A)

#### Climatic niche metrics

We quantified climatic niche metrics following the approach by Schweiger et al. (2014), from where climatic niche metrics were readily available for butterflies. For birds and moths, we obtained atlas data for Europe (Supplementary Text S3) and calculated the niche metrics following the same approach as Schweiger et al. (2014). The Europe-wide atlas data were overlaid on the CGRS grid (Common European Chorological Grid Reference System from the European Environment Agency). We used interpolated climate data on the same CGRS grid (originally developed in the ALARM project (Fronzek et al., 2012; Settele et al., 2005) and parameters summarized by Schweiger et al. (2014)) to calculate a variety of climatic niche metrics for each species based on the climate data from the grid cells where the species occurs in Europe. We also derived a metric of range size using this approach (number of occupied grid cells in Europe).

In this study, we chose the mean and standard deviation (SD) for mean annual temperature (MAT; corresponding to STI, Devictor et al., 2012) and growing degree days above 5 °C for January–August (hereafter GDD5) as candidate variables to describe the thermal niche. We chose the mean and SD for annual precipitation sum (PREC) and soil water content (SWC) of the upper horizon (0.5 m) as candidate variables to describe the moisture niche. The mean of each parameter thus describes the average conditions in which the species occurs, while the SD describes the absolute breadth of the niche. To account for relative variation in the climatic parameter compared to the mean, we also calculated the coefficient of variation (CV = SD/mean). This is meaningful since a large variation around a small mean tends to imply a more variable set of de facto conditions, since variable conditions closer to freezing temperatures or dry conditions can be more physiologically demanding. For calculating CV for MAT, the degrees Celsius were converted into Kelvin. Interrelationship of mean, SD, and CV for the for climatic parameters and four examples of underlying atlas data are shown in Supplementary Figure S3.

#### Data on ecological traits and habitat use

The trait variables used here included: body size (continuous variable), overwintering mode (for birds: resident; short-distance migrant, and long-distance migrant; for Lepidoptera: adult, larvae, pupa, egg), and number of generations or broods per season (two levels: one or less and two or more). These traits were chosen as they have been linked to species’ responses to climate and environmental change in previous empirical studies (Fourcade et al., 2021; Kluen et al., 2017; Laaksonen & Lehikoinen, 2013; Lehikoinen & Virkkala, 2016; Pöyry et al., 2009; Välimäki et al., 2016; WallisDeVries, 2014). Moreover, these traits are comparable for Lepidoptera and birds, and information about them is available for most of our studied species. We also tested for effect of range size across Europe. See Supplementary Text S4 for data sources and trait groupings.

### Analyses

#### Estimating shift in range boundaries

To estimate the magnitude and direction (southward or northward) of the northern range boundary shift between the two time periods, we used quantile regression (Koenker et al., 2017) as implemented in the *quantreg* package for R (Koenker, 2021). This regression method estimates conditional quantiles of a data distribution instead of a mean outcome. For butterflies and moths, quantile regression was fitted separately for each of the five subsets of the data (Supplementary Text S1), after which the estimates for the five outcomes, including 95% confidence intervals, were averaged to produce the final estimate. We estimated the effect that time period (a factor with two levels) had on the 0.75 and 0.9 quantiles of latitudes of distribution points for each species (Figure 1). The models were fitted separately for each species. We inferred the location of the 0.75 and 0.9 perimeter in T_1_ as the estimated intercept. The location of corresponding perimeters in T_2_ was arrived at by adding the estimated difference between the two levels of the time period categorical variable to the intercept.

#### Analyses of niche metric and trait effects on range shifts

We used a weighted linear regression to test the relationship between species range shifts and niche metrics and traits. The shift in range boundaries (i.e., kilometers change in quantile) was used as a continuous response variable and we fitted models separately for each taxonomic group. We used eight explanatory variables: four climatic variables (mean thermal niche, breadth of thermal niche, mean moisture niche, and breadth of moisture niche), three trait variables (overwintering mode, number of generations or broods per season, and body size), and range size across Europe. Continuous covariates were scaled and centered. We applied weights defined as the inverse of the confidence interval (95%) of the estimated range shift to inform the model of uncertainty related to the estimated shift. For the main models (one for each taxonomic group), we chose to use the change in the 0.9 quantile to describe range shifts. We used the mean and SD of MAT to describe the thermal niche, since MAT reflects thermal conditions also outside of the growing season. We chose SWC to describe the moisture niche since the mean and SD of PREC correlated strongly with each other (Supplementary Figure S6). Our main model therefore tested the effect of mean and SD of MAT and SWC, and traits on the 0.9 quantile. We also applied three alternative models to understand whether the results were dependent on the chosen variables. These alternative models were: (a) the effect of mean and SD of MAT and SWC and traits on the 0.75 quantile (i.e., as opposed to the 0.9 used in the main model), (b) the effect of mean and CV (relative niche breadth; as opposed to SD which describes the absolute niche) of MAT and SWC and traits on the 0.9 quantile, and (c) the effect of mean and SD of GDD and PREC and traits (as opposed to MAT and SWC on the 0.9 quantile). The results for the alternative models are presented in Supplementary Text S5 and Supplementary Tables S3–S6.

We used the *ols_step_best_subset* function in the *olsrr* package (Hebbali, 2020) to fit and compare models with all possible combinations of our eight explanatory variables (2^8^ = 256 potential models). This method provides a list of the best fitting models for models with one, two, three … 8 variables based on several criteria like R^2^, Mallow’s Cp, and AIC (Hebbali, 2020) (Supplementary Table S2). From this priority list, we chose the best explaining model based on the lowest AIC value (Akaike’s Information Criterion; Burnham & Anderson, 2004) while also considering increase in adjusted and predicted R^2^. However, in order to choose a less parsimonious model (a model with more estimated parameters) the AIC was required to be at least two units lower, since models with less than two-unit difference are considered to have the same information value (Burnham & Anderson, 2004; Symonds & Moussali, 2011). Based on this model selection process, we determined a minimal model that fits the data best and identified the variables that help explain observed range shifts. The explanatory power of the variables in the final models was assessed by interpreting the summary table and comparing the AIC of models where one of the variables had been left out using the *drop1* function in R (Table 2). We assessed model performance of the final models by both visualizing and testing normality and heteroscedasticity of model residuals, collinearity (variance inflation factors), and influential outliers using the *performance* package in R (Lüdecke et al., 2021; see Supplementary Figure S8 for the main model and Supplementary Text S5 for the alternative models).

**Table 1.** Results of the best subsets regression comparing all possible combinations of our eight explanatory variables.

	Predictors	Predictors	Adj. R-Square	Pred. R-Square	R-Square	AIC
a)	Best subsets regression for moths					
	Tbreadth	1	0.0744	0.0705	0.0587	2911.2071
	Tmean + Tbreadth	2	0.1438	0.1365	0.1227	2894.5814
	**Tmean + Tbreadth + Wintering**	**3**	**0.1872**	**0.1697**	**0.1491**	**2888.1533**
	Tmean + Tbreadth + Wintering + Numb. Gen.	4	0.1954	0.1746	0.1505	2887.7337
	Tmean + Tbreadth + Wintering + Numb. Gen. + Size	5	0.2034	0.1792	0.1521	2887.3376
	Tmean + Tbreadth + Wintering + Numb. Gen. + Size + Range size	6	0.2054	0.1778	0.1463	2888.7340
	Tmean + Tbreadth + Mmean + Wintering + Numb. Gen. + Size + Range size	7	0.2066	0.1754	0.1403	2890.3699
	Tmean + Tbreadth + Mmean + Mbreadth + Wintering + Numb. Gen. + Size + Range size	8	0.2072	0.1725	0.1333	2892.1833
b)	Best subsets regression for birds					
	Wintering	1	0.0547	0.0321	−0.02	992.2917
	Tbreadth + Wintering	2	0.1230	0.0913	0.0272	987.7673
	**Tbreadth + Wintering + Numb. Gen.**	**3**	**0.1877**	**0.1480**	**0.0759**	**983.1005**
	Tbreadth + Wintering + Numb. Gen. + Size	4	0.1998	0.1505	0.0304	983.7848
	Tbreadth + Wintering + Numb. Gen. Size + Range size	5	0.2153	0.1564	0.0393	984.0905
	Tbreadth + Mmean + Wintering + Numb. Gen. + Size + Range size	6	0.2189	0.1497	0.0302	985.6845
	Tmean + Tbreadth + Mmean + Wintering + Numb. Gen. + Size + Range size	7	0.2238	0.1442	0.014	987.1365
	Tmean + Tbreadth + Mmean + Mbreadth + Wintering + Numb. Gen. + Size + Range size	8	0.2249	0.1343	−0.0129	989.0148
c)	Best subsets regression for butterflies					
	**Mbreadth**	**1**	**0.0751**	**0.0583**	−**0.0334**	**673.4953**
	Tbreadth + Wintering	2	0.1446	0.0788	−0.0531	675.0447
	Mbreadth + Wintering + Numb. Gen.	3	0.1772	0.0965	−0.0708	674.8316
	Tbreadth + Mbreadth + Wintering + Numb. Gen.	4	0.1920	0.0951	−0.0912	675.7916
	Tbreadth + Mbreadth + Wintering + Numb. Gen. + Range size	5	0.1947	0.0797	−0.1331	677.6021
	Tmean + Tbreadth + Mmean + Mbreadth + Wintering + Numb. Gen.	6	0.2044	0.0718	−0.197	678.9105
	Tmean + Tbreadth + Mmean + Mbreadth + Wintering + Numb. Gen. + Range size	7	0.2056	0.0534	−0.253	680.8292
	Tmean + Tbreadth + Mmean + Mbreadth + Wintering + Numb. Gen. + Size + Range size	8	0.2057	0.0330	−0.2993	682.8228

This method provides a list of the best fitting models for models with one, two, three … 8 variables based on several criteria like R^2^, Mallow’s Cp, and AIC (Hebbali 2020) (Supplementary Table S2). Tmean = mean thermal niche; Tbreadth = breadth of thermal niche; Mmean = mean moisture niche; Mbreadth = breadth of moisture niche; Wintering = overwintering mode; Numb. Gen. = number of generations or broods per season; Size = body size; Range size = range size across Europe. From this priority list, we chose the best explaining model based on the lowest AIC value (Akaike’s Information Criterion; Burnham & Anderson 2004) while also considering increase in adjusted and predicted R^2^. However, in order to choose a less parsimonious model (a model with more estimated parameters) the AIC was required to be at least two units lower, since models with less than two-unit difference are considered to have the same information value (Burnham & Anderson 2004; Symonds & Moussali 2011). Based on this model selection process, we determined a minimal model that fits the data best and identified the variables that help explain observed range shifts.

**Table 2.** Summary table full models and variable omission.

	Summary table					Variable omission				
	Parameter	Estimate	Std. Error	t value	Pr(≥|t|)	Variable dropped	ΔDf	ΔSum of Sq	RSS	AIC
Moth	(Intercept)	12.327	25.631	0.481	0.631014	<none>			7726.1	844.74
	scale(Tmean)	33.812	8.611	3.927	**0.000114**	scale(Tmean)	1	515.67	8241.7	**858.18**
	scale(Tbreadth)	−30.489	6.432	−4.740	**3.74e-06**	scale(Tbreadth)	1	751.48	8477.5	**864.92**
	scale(Mmean)	−3.053	8.854	−0.345	0.730535	scale(Mmean)	1	3.98	7730.0	842.86
	Wintering—egg	22.632	28.433	0.796	0.426857	Wintering	3	496.61	8222.7	**853.63**
	Wintering—larva	43.360	27.444	1.580	0.115491					
	Wintering—pupa	−6.544	27.463	−0.238	0.811872					
Bird	(Intercept)	13.514	9.309	1.4517	0.15039	<none>			1444.5	254.44
	scale(Tbreadth)	−21.557	9.840	−2.1908	**0.03131**	scale(Tbreadth)	1	112.964	1557.5	**258.99**
	scale(Mbreadth)	2.305	5.625	0.4097	0.68306	scale(Mbreadth)	1	1.457	1446.0	252.53
	WinteringR	48.225	19.640	2.4554	**0.01619**	Wintering	2	136.469	1581.0	**258.29**
	WinteringS	15.158	17.504	0.8660	0.38902					
Butterfly	(Intercept)	33.593	9.987	3.364	0.00147	<none>			1280.8	185.39
	scale(Tbreadth)	1.322	11.181	0.118	0.90632	scale(Tbreadth)	1	0.351	1281.1	183.41
	scale(Mbreadth)	13.939	11.808	1.180	0.24329	scale(Mbreadth)	1	34.996	1315.8	184.93
	scale(range.size)	20.347	13.094	1.554	0.12639	scale(range.size)	1	60.641	1341.4	**186.03**

In bold statistically significant effects (<0.05) of variables according to estimated t-values (to the left) and AIC values that indicate decreased model fit if variable is dropped (ΔAIC >2; to the right). Mean thermal niche was measured as the average of MAT across the species distribution, thermal niche breadth as the SD of MAT, and moisture niche breadth as the SD of SWC.

R^2^ = 0.255, 0.132, 0.128 and adjusted R^2^ = 0.235, 0.09, 0.77 for moth, birds, and butterflies, respectively.

All data management and analyses were conducted in the R environment (R studio; version 4.0.5; R Core Team, 2022).

## Results

On average, species shifted their northern range boundary (0.9 quantile) 29.2 km northwards during the study period. The mean shift was highest for butterflies (43.1 km), followed by birds (33.2 km), and moths (24.4 km). When accounting for the time between the study periods (for birds): T1 = 1974–1989 (mid-year = 1981.5), T2 = 2006–2010 (mid-year = 2008); for moths and butterflies: T1= 1992–1996 (mid-year = 1994), T2 = 2013–2017 (mid-year = 2015), butterflies, moths, and birds had moved northwards, on average, with a speed of 1.95, 1.11, and 1.25 km/year, respectively.

**Figure 2.**
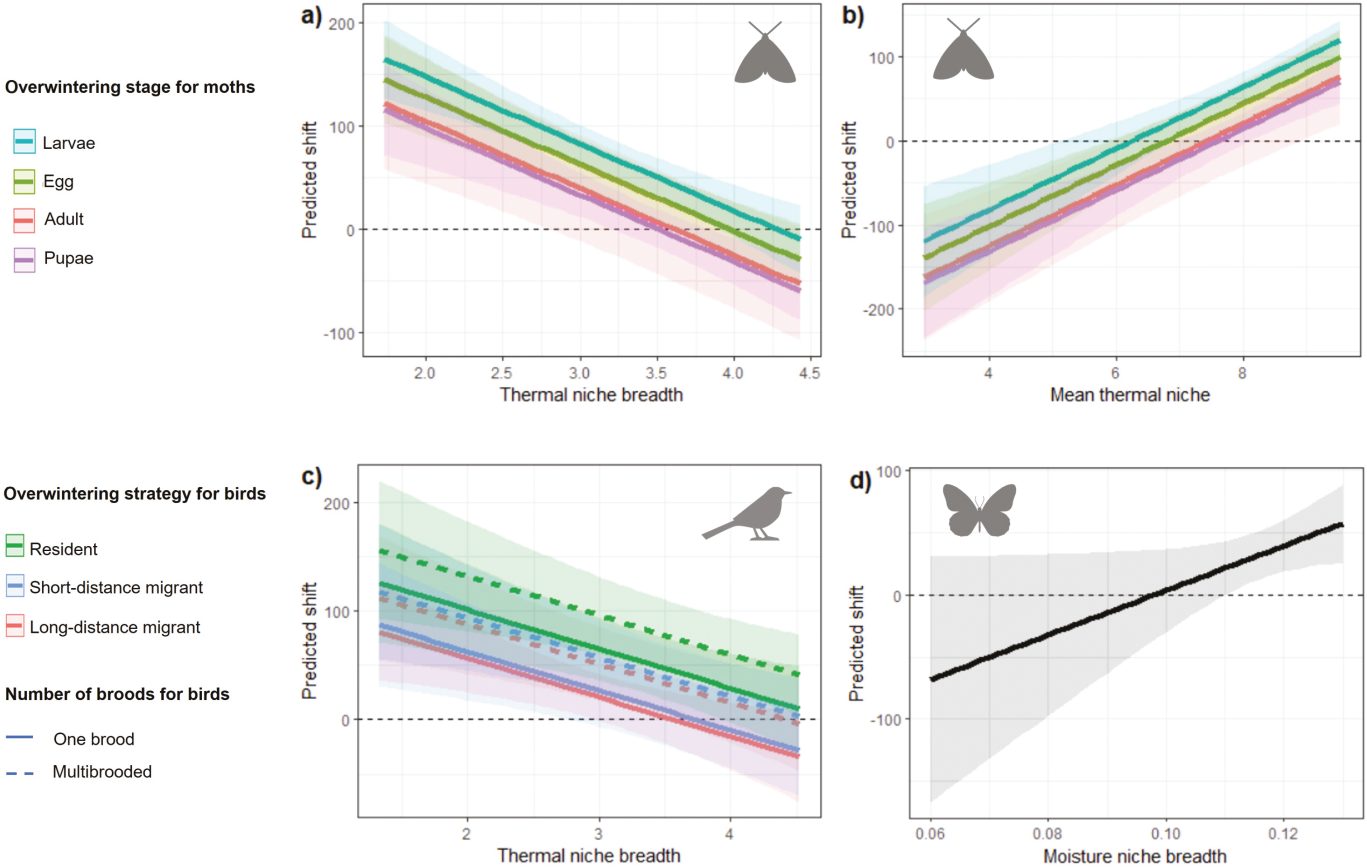
Predicted shift (kilometers) in the northern range boundary as a function of influential variables included in the final model. (A and B) moths; (C) birds; and (D) butterflies. For each continuous variable in the final model, we produced a new data set ranging from the minimum to maximum values of that variable, while keeping all other continuous variables at their mean values. We then predicted the range shift based on these new data, accounting for all levels of categorical variables included in the final model. The thermal niche breadth was significantly negatively connected with a northward shift for moths (A) and birds (C), implying that species with a smaller relative thermal niche shifted further northwards. Moths with a warmer mean thermal niche also tended to shift further north (B). Butterflies showed a tendency towards the opposite regarding niche breadth, with a relatively broad moisture niche (D) connected with a northward shift. Overwintering mode improved model fit for both moths and birds. Moths overwintering as larvae and eggs shifted their ranges more northwards than those overwintering as adults or pupae. For birds, we also found evidence that including wintering mode, i.e., migratory behavior, improved model fit. Range edges of resident birds moved significantly further north than that of long-distance migrants. As the short and long-distance migrants’ estimates did not differ significantly from each other, it is likely that resident birds moved further northwards than short-distance migrants too. Including number of broods per year in the bird model provided weak support for a better model fit, with birds producing multiple broods per year showing stronger northward shifts. None of the candidate traits improved model fit for butterflies.

The final model for all species groups contained a climatic niche metric describing niche breadth. The thermal niche breadth was related with a smaller northward shift for moths and birds, with species with narrow absolute thermal niches tending to move more northwards (Tables 1 and 2; Figure 2). The moisture niche breadth had a positive connection with butterfly range shift, as species with a broader moisture niche tended to move further north. The average thermal niche had a positive relationship with northward shifts for moths (Table 2; Figure 2), indicating that moths occupying warmer niches, in addition to those with narrower niches, moved northwards more strongly.

Overwintering mode improved model fit for both moths and birds. Moths overwintering as larvae and eggs shifted their ranges more northwards than those overwintering as adults or pupae. For birds, including wintering mode, i.e., migratory behavior, improved model fit. Range edges of resident birds moved significantly further north than that of long-distance migrants. Moreover, as the short and long-distance migrants’ estimates did not significantly differ from each other, it is likely that resident birds moved further northwards than short-distance migrants too. Including the number of broods per year in the bird model provided weak support for an improved model fit, with birds producing multiple broods per year showing stronger northward shifts. None of the included traits improved model fit for butterflies as measured by AIC. However, it is noteworthy that the increase in adjusted R^2^ indicates some potential explanatory power by wintering mode and number of generations per year also for butterflies (see Table 1).

Model performance checks of the main model revealed no issues with normality of residuals, heterogeneity, collinearity, or outliers (Supplementary Figure S8). See Supplementary Figure S7 for scatter plots and univariately fitted regression lines for the connection between each candidate variable and shift in the 0.9 quantile. The results of the alternative models were largely qualitatively consistent with the main model (see Supplementary Text S5; Supplementary Tables S3–S6).

## Discussion

We found that birds and moths with more narrow thermal niches and butterflies with broader moisture niches tended to shift their northern range border northward. Moths that are adapted to warmer conditions were also more likely to expand their range edges towards the north compared to species with an affinity to cooler conditions. Species with a wider thermal niche likely have greater tolerance for variation in thermal conditions. A wide tolerance for a species may reflect high tolerance for each individual, high intrapopulation variation in tolerance, or high interpopulation variation in tolerance across space (cf. Figure 1 in Bolnick et al., 2007). A high thermal tolerance at any of these scales can help species to cope or adjust in place either through local responses or intraspecific gene flow. The exact mechanistic process cannot be validated with a macroecological approach used in this study, but our findings nevertheless point to a potential connection that warrants further study (cf. Hoffman et al., 2003). Such studies could shed light on the mechanism through which past evolutionary processes, which have formed a continuum of species that differ in their climatic tolerance, may currently dictate how species respond to rapid anthropogenic climate change.

Our results for moths and birds provides support for the hypothesis that species with relatively narrow thermal niches tend to move along with the shifting thermal isoclines. This suggests the potentiality for lower degree of thermal tolerance and perhaps less phenotypic plasticity to varying climates to predict a stronger need to move as climate changes in these two species groups. Shifting ranges together with a changing climate of course requires that individuals can actively seek out suitable area to colonize or have a high intrinsic dispersal tendency around their birth site (Pöyry et al., 2009; Warren et al., 2001), are relatively good dispersers, and can find otherwise suitable conditions and resources within the new, climatically suitable, landscapes. Evolution of increased dispersal may also occur rapidly as the selection pressure for dispersal would be higher for populations with a higher proportion of optimal conditions outside of their original location (Bridle et al., 2014; Saastamoinen et al., 2018; Travis et al., 2013), which would more likely be the case for a species with a narrow tolerance, assuming that individual tolerance resembles the species-wide tolerance. Based on this study, we can only speculate on the dispersal ability of the species that are not shifting their range edges, but we can assume that species with narrower niches likely have evolved to specialize on certain conditions wherefore a relatively high dispersal rate and ability to actively seek out suitable environments could have evolved hand-in-hand (Jacob et al., 2018). Species with a wider thermal tolerance, on the other hand, could use their flexibility for coping with novel or rapidly changing climatic conditions in situ, both if individuals possess higher tolerance towards climatic conditions and if (random) colonization events within the species range introduce individuals with a more suitable adaptation. Instead of these generalists being able to colonize novel area more readily than specialists, long-term evolution may have included a trade-off between evolvability in physiological traits and habitat-seeking ability (Jacob et al., 2018).

The tendency for the niche breadth effect to be mediated by the average thermal niche (=STI; Devictor et al., 2012; Figure 2) for moths is in line with previous studies showing that more southerly distributed species tend to expand northwards more prominently (Brommer et al., 2012; Hitch & Leberg, 2007; Parmesan, 2006; Thomas & Lennon, 1999). In our data, we also saw indications that species, especially moths, with a lower northern range edge at T_1_ tended to move further north (Figure 1D and Supplementary Figure S4). This could be an indication of species distributed further to the south having more geographical space to disperse into and thus more successfully colonizing new area. However, this tendency may be an artifact of the niche breadth effect correlating with thermal tolerance as measured either through the latitudinal position of the range edge at T_1_ or the mean thermal niche. Rapoport’s rule (Stevens, 1989) states that species at higher latitudes would tend to have broader ranges (and thus potentially broader niches) than species occurring closer to the equator. Although this theory has been contested, it seems to hold at least on the regional level and across continental Eurasia (Addo-Bediako et al., 2000; Rohde, 1996; Ruggiero & Werenkraut, 2007). Thus, if species occurring in warmer climates do tend to have narrower niches, using the average thermal niche to explain range shifts could, at least partially, act as a proxy for niche breadth and thereby be confused as playing a proximate role in climate change responses.

For butterflies, the moisture niche breadth was informative for explaining range shift, but not for birds and moths. A study on birds in Sweden found that temperature was more important for birds than precipitation when tracking suitable climates (Tayleur et al., 2015). Other studies have found that long-term change in precipitation can have a larger effect on range shifts than temperature, and that moisture availability can affect species performance and their distribution (Boyle et al., 2020; Kahilainen et al., 2018; MacLean & Beissinger, 2017; Riddell et al., 2017). In Finland, with high prevalence of permanent freshwater sources (lakes, rivers, wetlands) and annual rainfall of 500–700 mm, moisture is likely not a restrictive factor per se. Precipitation has been predicted and observed to increase in northern Europe but this change is not correlated with latitude in the same manner as temperature (Ruosteenoja & Jylhä, 2021). As temperature increases, evaporation also increases which in turn can even increase the incidence of extreme drought events. Indeed, long-term dry spells have reportedly resulted in substantial declines in butterfly populations (van Bergen et al., 2020). Interannual variation in moisture availability is probably more decisive in determining local population dynamics (Mills et al., 2017), but it has the potential of affecting also broad-scaled range shifts (Oliver et al., 2015). Given this, the effect of precipitation change would not necessarily manifest as northwards range shifts, which was measured here. Nevertheless, we found indications of moisture niche breadth, more specifically SWC, affecting butterflies. Thus, the effects of changes in moisture conditions and variability on butterflies warrants further studies.

Butterflies did not show the same pattern of increased ranges shifts for more narrow climatic niches as birds and moths. They also differed in showing the largest on average shifts northwards (see also Pöyry et al., 2018). In addition, a broader moisture niche increased the tendency for northwards shifts for butterflies. This is in line with the findings by Pöyry et al., (2009) who concluded that generalist butterflies in Finland had shifted their ranges more than specialist species, the latter which may have difficulties moving across the landscape through suitable habitats. Habitat availability may indeed explain the lack of expected northward expansions for butterflies (Oliver et al., 2017; Platts et al., 2019; Warren et al., 2001). In Finland, the occurrence of several butterflies is heavily limited by habitat availability, particularly those that rely on cultural or peatland habitats, which have declined in both quantity and quality over that past decades (Kuussaari et al., 2007). Thus, any range expansion effect that could otherwise occur may be diluted by the lack of suitable habitat in the landscape (Angert et al., 2020; Kwon et al., 2021) or because other axes of the niche, like resources and biotic interactions (Carscadden et al., 2020), or novel abiotic conditions (Spence & Tingley, 2020) not captured by commonly used climatic parameters act in a restricting fashion. Another driver behind the deviating results for butterflies compared to the other ectothermic group studied here (moths) could be the different ways that temperature increase affects diurnal and nocturnal ectotherms, as night-time temperatures have increased more than day-time temperatures (Speights et al., 2017; Tuomenvirta et al., 2000).

Of the ecological traits, only few significant relationships were observed. For both moths and birds, wintering mode had explanatory power, while for butterflies we found no statistically supported relationships between traits and range shifts (Table 2). This finding is in line with previous meta-analyses, reporting a lack of explanatory power using morphological and life-history traits (Angert et al., 2020; Beissinger & Riddell, 2021; Buckley & Kingsolver, 2012; MacLean & Beissinger, 2017). Range size did not explain variation in the data better than the climatic niche breadth for any of the three studied species groups. Thus, although range size and climatic niche breadth may be correlated (see, e.g., Dallas & Kramer, 2022; Slatyer et al., 2013), range size itself may not be helpful as a predictor of range shifts under contemporary climate change.

Recent studies comparing two main measurable adjustments to climate change, phenology, and range shifts (Amano et al., 2014; Hällfors et al., 2021; Socolar et al., 2017), have found that most studied species tend to either adjust in place through phenology shifts or shift their ranges. These findings rely on the same hypothesis as tested here, that species with a broader tolerance towards climatic conditions may adjust in place and remain within their current distributions (Carscadden et al., 2020), while species that are not able to adjust experience a stronger pressure to move across space and thus would tend to make better use of this strategy (Beissinger & Riddell, 2021; Herrera et al., 2018; Reif & Flousek, 2012). Our study provides support for this hypothesis through evidence on the importance of niche breadth and thereby climatic tolerance as a means to cope with environmental change. On a more general level, this suggests that past evolutionary processes that have formed a continuum of species that differ in their climatic niche breadth (Lancaster, 2022), may currently dictate how species respond to rapid anthropogenic climate change. Species’ flexibility vs. selectivity for climatic conditions may thus provide a key to understanding both observed and future range shifts.

## Supplementary Material

qrad004_suppl_Supplementary_Material

## Data Availability

Data used to conduct the analyses are available in Dryad: https://doi.org/10.5061/dryad.z8w9ghxh7
