# Supplementary material for: Recent range shifts of moths, butterflies, and birds are driven by the breadth of their climatic niche
Source: Evol Lett. 2023 Mar 12;8(1):89–100. doi: 10.1093/evlett/qrad004 (PMC10872046; doi:10.1093/evlett/qrad004)
Supplement: qrad004_suppl_Supplementary_Material [file qrad004_suppl_supplementary_material.pdf]

Supplementary materials for

Hällfors M.H., Heikkinen R.H., Kuussaari M., Lehikoinen A., Luoto M., Pöyry J., Virkkala R., Saastamoinen M., and Kujala H. (2023). Recent range shifts of moths, butterflies, and birds are driven by the breadth of their climatic niche. *Evolution Letters*, accepted January 29th 2023.

Data availability: Hällfors, Maria et al. (2023), Data for: Recent range shifts of moths, butterflies, and birds are driven by the breadth of their climatic niche, Dryad, Dataset, https://doi.org/10.5061/dryad.z8w9ghxh7

Contents:

Text S1-S5
Figures S1-S8
Tables S1-S6

Text S1. Distribution data and delimitation

We started off with a selection of 244 moth and 91 butterfly species. These moths and 45 of the butterfly species were selected for a previous study on phenology and range shifts based on the availability of adequate numbers of systematically collected monitoring and trap dates (Hällfors *et al.*, 2021). Although we do not use those moth trap data in this study, we opted for using the same moth species here as in Hällfors et al. (2021) allowing direct comparisons between the studies, and as this set covers the most common and abundantly occurring species in Finland. The lepidoptera species in this study cover almost 80% of the butterfly species ever observed in Finland and circa 27% of moths commonly monitored in Finland. We excluded five butterfly species and one moth species that are migratory and do not have a permanent breeding population in Finland (*Pieris rapae*, *P. brassicae*, *Vanessa atalanta*, *V. cardui, Colias hyale*, and *Autographa gamma*), leaving us with 243 moth and 86 butterfly species at this stage. For these species, we sourced observations that were available in the Insect database and National Butterfly Monitoring Scheme (NAFI; Saarinen *et al.*, 2003), through the Finnish Biodiversity Information Facility (FinBIF) in December 2019 (moths) and June 2022 (butterflies) (Text S2). The data were divided into two five-year periods: 1992–1996 (hereafter T_1) and 2013–2017 (hereafter T_2) and converted into presence-only data for each 10 x 10 km grid square (119 621 observations). We excluded two moth and 23 butterfly species that had been observed in less than 20 grid cells in either one of the time periods (117 486 observations). The total number of presence squares at T_2 was substantially higher than at T_1 due to increased sampling effort over time. To account for the change in sampling effort, we divided the data into five latitudinal zones (Fig. S1) and randomly subsampled the pooled observations of all species in T_2 so that the number of observations in T_2 matched the number of observations in T_1 within the latitudinal zone. This was repeated five times leaving us with five subsets with a total of 78 380 observations in each for these 241 moth and 63 butterfly species. For a comparison of subsamples across the latitudinal zones, see Fig. S2.

For birds, we used distribution data on terrestrial breeding birds, sourced from three national bird atlases through the Finnish Natural History Museum (Text S2). These atlases have been compiled from national bird surveys carried out during 1974–1979, 1986–1989 and 2006–2010, respectively. The three atlases contain an index of breeding probability (ranging from 0 = not found; to 4 = confirmed breeding) for each bird species on a 10 x 10 km uniform grid that covers the entire area of Finland (3813 grid squares; (Väisänen *et al.*, 1998)). We took breeding probability of 0 (not found) to represent absence and all other classes to represent presences. As the third atlas has been surveyed more extensively in comparison to first two atlases, and following earlier practices, we used the pooled first and second atlas data, covering the time period 1974–1989 (hereafter T_1) and compared this to the third (2006–2010) atlas (hereafter T_2) to reduce potential observation biases due to differences in survey effort (Kujala et al. 2013; Virkkala & Lehikoinen, 2017). We excluded all waterfowl and species that had been observed breeding in less than 20 grid cells in either one of the time periods leaving us with 177 bird species.

Finally, we harmonized the distribution extents of the study species and included only species that likely have their northern range borders in Finland. In Finland, moths, butterflies, and breeding birds include several arctic and subarctic species that predominantly occupy the northern parts of the country and for which the leading distribution edge is close to or outside the country borders. Consequently, we removed two moth, six butterfly and 90 bird species for which the centre point of distribution in Finland in T_1 was ≥ 7 000 000 north in the Finnish uniform coordination system (63º4' N in degrees; range in Finland 59º46' – 70º5'N; Fig. S1) (Brommer, 2004; Brommer *et al.*, 2012; Kujala *et al.*, 2013). For birds, the centre point was

weighted by their breeding category in T_1. This allowed us to focus on the predominantly southern species with leading distribution edges in Finland. The consequent data used for the main analyses thus consisted of 383 species: 239 species of moths, 57 species of butterflies, and 87 species of birds for which the shift in the northern facets of distribution between two periods of time 17 years apart were measured.

References:

Brommer, J.E. (2004) The range margins of northern birds shift polewards. Annales Zoologici Fennici, 41, 391–397.

Brommer, J.E., Lehikoinen, A. & Valkama, J. (2012) The Breeding Ranges of Central European and Arctic Bird Species Move Poleward. PLOS ONE, 7, e43648.

Hällfors, M.H., Pöyry, J., Heliölä, J., Kohonen, I., Kuussaari, M., Leinonen, R., *et al.* (2021) Combining range and phenology shifts offers a winning strategy for boreal Lepidoptera. *Ecology Letters*, **24**, 1619–1632.

Kujala, H., Vepsäläinen, V., Zuckerberg, B. & Brommer, J.E. (2013) Range margin shifts of birds revisited – the role of spatiotemporally varying survey effort. *Global Change Biology*, **19**, 420–430.

Saarinen, K., Lahti, T. & Marttila, O. (2003) Population trends of Finnish butterflies (Lepidoptera: Hesperioidea, Papilionoidea) in 1991–2000. *Biodiversity & Conservation*, **12**, 2147–2159.

Väisänen, R.A., Lammi, E. & Koskimies, P. (1998) *Muuttuva pesimälinnusto*. Otava, Keuruu.

Virkkala, R. & Lehikoinen, A. (2017) Birds on the move in the face of climate change: High species turnover in northern Europe. *Ecology and Evolution*, **7**, 8201–8209.

Text S2. Data license

Distribution data on Lepidoptera

Observational data on Lepidoptera, available through the Insect database and National Butterfly Monitoring Scheme (NAFI; Saarinen et al. 2003), were sourced from the Finnish Biodiversity Information Facility (FinBIF). For moths, the data sourcing from FinBIF was conducted in December 2019 on the superfamily or species level and the following batches were downloaded: http://tun.fi/HBF.38384, http://tun.fi/HBF. 38387 , http://tun.fi/HBF. 38386 , http://tun.fi/HBF. 38404. 1 858 745 observations on the 244 moth species were thereafter separated from the other species within the R environment. For butterflies, data on the 91 species was directly sourced into R using the FinBIF R package (Morris 2020) in June 2022, yielding a total of 768 027 observations.

Distribution data on birds

Results of the 1st, 2nd and 3rd Finnish bird atlas. Finnish Museum of Natural History, University of Helsinki (Luomus). Used with Creative Commons Attribution 4.0 -license. http://atlas3.lintuatlas.fi/english/

Reference:

Morris W (2022). Introduction to the finbif package. doi:10.5281/zenodo.3612814, R package version 0.7.1, https://luomus.github.io/finbif/articles/finbif.html.

Text S3. Data used to arrive at climatic niche metrics.
For butterflies, we employed metrics provided by Schweiger et al. (2014), based on the distribution atlas on butterflies in Europe. For birds, we used distribution data from BirdLife International (BirdLife International, 2020). These data are based on a variety of sources and give different levels of site occupancy. We included the location where each species is extant or probably extant, native or reintroduced, and known or thought very likely to be resident or occur during the breeding season. For moths we used available digitized atlas information on geometric moths from Heidrich *et al.*, (2018) which are based on *The Geometric Moths of Europe volumes 1–4* (Hausmann, 2001, 2004; Mironov, 2003; Hausmann & Viidalepp, 2012; Hausmann *et al.*, 2012.) and digitized atlas maps on 176 other species based on printed atlases (Fibiger 1990, 1993, 1997, 2009, Fibiger et al. 1995, 2007, 2010; de Freina & Witt, 1987, 1990; Gouter et al. 2003; Hacker et al. 2002; Müller et al. 2019; Ronkay et al 1994, 2001; Skou & Sihvonen, 2015; Zilli et al. 2005). The atlas data were overlaid on the CGRS grid (Common European Chorological Grid Reference System from the European Environment Agency). We used interpolated climate data on the same CGRS grid (originally developed in the ALARM project (Settele *et al.*, 2005; Fronzek *et al.*, 2012) and parameters summarized by Schweiger et al. (2014)) to calculate climatic niche metrics for each species. Interrelationship of climatic niche metrics and four examples of atlas data in Fig. S3.

References:

BirdLife. (2020) *BirdLife International and Handbook of the Birds of the World (2020) Bird species distribution maps of the world*.

Fibiger, M. (1990). *Noctuidae Europaeae 1: Noctuinae 1*. Sorø, Denmark: Entomological Press.

Fibiger, M. (1993). *Noctuidae Europaeae 2: Noctuinae 2*. Sorø: Entomological Press.

Fibiger, M., Hacker, H., & Goater, B. (1995). *Noctuidae Europaeae: 7*. Sorø: Entomological Press.

Fibiger, M. (1997). *Noctuidae Europaeae, Vol. 3: Noctuinae III*. Sorø: Entomological Press.

Fibiger, M., Hacker, H., & Ronkay, L. (2007). *Noctuidae Europaeae: Amphipyrinae, Condicinae, Eriopinae, Xyleninae (part)* (Vol. 9). Sorø: Entomological Press.

Fibiger, M. (2009). *Noctuidae Europaeae: Pantheinae, Dilobinae, Acronictinae, Eustrotiinae, Nolinae, Bagisarinae, Acontiinae, Metoponiinae, Heliothinae, and Bryophilinae (Vol. 11).* Sorø: Entomological press.

Fibiger, M., Ronkay, L., Yela, J. L., & Zilli, A. (2010). Noctuidae Europaeae. Volume 12: Rivulinae, Boletobiinae, Hypenodinae, Araeopteroninae, Eublemminae, Herminiinae, Hypeninae, Phytometrinae, Euteliinae and Micronoctuidae including supplement to Volume 1–11. *Noctuidae Europaeae*. Sorø: Entomological Press.

Fibiger, M., Thomas, J., László, , & Tibor Csóvári. (2011). *Noctuidae Europaeae: Including phylogeny and check list of the Quadrifid Noctuoidea of Europe.* Sorø: Entomological Press.

Freina, J.J. de & Witt, T.J. (1987) *Die Bombyces und Sphinges der Westpalaearktis (Vol 1. Insecta, Lepidoptera).* Edition Forschung u. Wissenschaft.

Freina, J. J. de & T. J. Witt. 1990. *Die Bombyces und Sphinges der Westpalaearktis (Insecta, Lepidoptera). Band 2. Cossoidea, Hepialoidea, Pyraloidea, Zygaenoidea.* – Edition Forschung & Wissenschaft, München. 134 pp., 10 pls.

Fronzek, S., Carter, T.R. & Jylhä, K. (2012) Representing two centuries of past and future climate for assessing risks to biodiversity in Europe. *Global Ecology and Biogeography*, **21**, 19–35.

Goater, B., Fibiger, M., & Ronkay, L. (2003). *Noctuidae Europaeae: Volume 10.* Sorø, Denmark: Entomological Press.

Hacker, H., Hreblay, M., & Ronkay, L. (2002). *Hadeninae I. Noctuidae europaeae 4.* Sorø: Entomol. Press.

Hausmann, A. (2001) *Introduction, Archiearinae, Orthostixinae, Desmobathrinae, Alsophilinae, Geometrinae. In A. Hausmann (Ed.), The geometrid moths of Europe 1.* Apollo Books, Stensrup, Denmark.

Hausmann, A. (2004) *Sterrhinae. In A. Hausmann (Ed.), The Geometrid Moths of Europe 2.* Apollo Books, Stensrup, Denmark.

Hausmann, A., Mironov, V., Sihvonen, P., Skou, P. & Viidalepp, J. (2014) *The Geometrid Moths of Europe (update).*

Hausmann, A. & Viidalepp, J. (2012) *Larentiinae I. In A. Hausmann (Ed.), The Geometrid Moths of Europe 3.* Apollo Books, Stensrup, Denmark.

Heidrich, L., Friess, N., Fiedler, K., Brändle, M., Hausmann, A., Brandl, R., *et al.* (2018) The dark side of Lepidoptera: Colour lightness of geometrid moths decreases with increasing latitude. *Global Ecology and Biogeography*, **27**, 407–416.

Müller, B., Erlacher, S., Hausmann, A., Rajaei, H., Sihvonen, P., & Skou, P. (2019). The Geometrid Moths of Europe, vol. 6, Ennominae II. Leiden: Brill.

Ronkay, G., & Ronkay, L. (1994). *Noctuidae Europaeae: Volume 6.* Sorø, Denmark: Entomological Press.

Ronkay, L., Hreblay, M., & Yela, J. L. (2001). *Noctuidae Europaeae: Volume 5*. Sorø, Denmark: Entomological Press.

Schweiger, O., Harpke, A., Wiemers, M. & Settele, J. (2014) CLIMBER: Climatic niche characteristics of the butterflies in Europe. *ZooKeys*, 65–84.

Settele, J., Hammen, V., Hulme, P., Karlson, U., Klotz, S., Kotarac, M., *et al.* (2005) ALARM: Assessing LArge-scale environmental Risks for biodiversity with tested Methods. *GAIA - Ecological Perspectives for Science and Society*, **14**, 69–72.

Skou, P. & Sihvonen, P. (2015) *The Geometrid Moths of Europe, Volume 5 Ennominae I*. E J Brill.

Zilli, A., Ronkay, L., & Fibiger, M. (2005). *Noctuidae Europaeae: Volume 8.* Sorø: Entomological Press.

Text S4. Data sources for traits, habitat, and range size

Trait data on birds were based on Solonen (1985) and Cramp *et al.* (1994) while data on Lepidoptera were collated from several sources (Mikkola & Jalas, 1977, 1979; Mikkola *et al.*, 1985, 1989; Marttila *et al.*, 1991, 1996; Jalas, 1992; Silvonen *et al.*, 2014; Middleton-Weilling et al. 2020). Size was measured as the total wingspan (in mm) of females for moths, as wing index for butterflies, and as mean mass for birds. Bird species that are multi-brooded in southern Finland and single-brooded in northern Finland were categorized as having two or more broods. Voltinism in Lepidoptera were combined into two levels: Semi- and univoltine species = one or less, and multivoltine species (including bivoltine species) = two or more (Pöyry *et al.*, 2017). See Table S1 for number of species in categorical traits groups. From the distributional data used for calculating climatic niche metrics, we also derived another ecological attribute: range size.

References:

Cramp, S., Simmons, K.E.L. & Perrins, C.M. (Eds.). (1994) *Perrins, Handbook or the Birds of Europe, the Middle East and North Africa: Birds of the Western Palearctic*. Oxford University Press, Oxford.

Jalas, I. (1992) *Perhostenkeräilijän opas [Lepidopterist's guide]*. 3rd edn. Otava, Keuruu.

Marttila, O., Haahtela, T. & Aarnio, H. (1991) *Suomen päiväperhoset. [Finnish Lepidoptera: Papilionoidea, in Finnish]*. 2nd edn. Kirjayhtymä, Helsinki.

Marttila, O., Saarinen, K., Haahtela, T. & Pajari, M. (1996) *Suomen kiitäjät ja kehrääjät. [Finnish Lepidoptera: Sphingidae, Lasiocampidae, Endromidae, Lemoniidae, Saturniidae, Notodontidae, Lymantriidae, Arctiidae, Nolinae, Dilobinae, in Finnish].* Kirjayhtymä, Helsinki.

Middleton-Welling, J., Dapporto, L., García-Barros, E. et al. A new comprehensive trait database of European and Maghreb butterflies, Papilionoidea. *Sci Data* **7**, 351 (2020). https://doi.org/10.1038/s41597-020-00697-7

Mikkola, K. & Jalas, I. (1977) *Suomen perhoset, yökköset 1. [Finnish Lepidoptera: Noctuidae 1, in Finnish].* Otava, Keuruu.

Mikkola, K. & Jalas, I. (1979) *Suomen perhoset, yökköset 2. [Finnish Lepidoptera: Noctuidae 2, in Finnish]*. Otava, Keuruu.

Mikkola, K., Jalas, I. & Peltonen, O. (1985) *Suomen perhoset, mittarit 1. [Finnish Lepidoptera: Geometroidea 1, in Finnish].* Suomen Perhostutkijain Seura, Tampere.

Mikkola, K., Jalas, I. & Peltonen, O. (1989) *Suomen perhoset, mittarit 2. [Finnish Lepidoptera: Geometroidea 2, in Finnish].* Recallmed, Suomen Perhostutkijain Seura, Hanko.

Pöyry, J., Carvalheiro, L.G., Heikkinen, R.K., Kühn, I., Kuussaari, M., Schweiger, O., *et al.* (2017) The effects of soil eutrophication propagate to higher trophic levels. *Global Ecology and Biogeography*, **26**, 18–30.

Silvonen, K., Top-Jensen, M. & Fibiger, M. (2014) *Suomen päivä- ja yöperhoset - maastokäsikirja* (*A field guide to the butterflies and moths of Finland) [In Finnish].* Bugbooks, Oestermarie

Solonen, T. (1985) *Suomen Linnusto: esiintyminen ja perusbiologiaa.* Lintutieto.

Text S5. Alternative models.

In addition to the main model testing the effect of mean and SD of MAT and mean and SD of SWC on a shift in the 0.9 quantile, we tested these three alternative models on each taxonomic group to assess the robustness of our results particularly in relation to alternative metrics for or range shift, niche breadth, and the thermal and moisture niche. These alternative models were:

1) the effect of mean and SD of MAT and SWC and traits on the 0.75 quantile (i.e. the same model as the main model, but for shift in the 0.75 quantile as opposed to the 0.9 used in the main model),

2) the effect of mean and CV (relative niche breadth, as opposed to SD which describes the absolute niche) of MAT and SWC and traits on the 0.9 quantile, and

3) the effect of mean and SD of GDD and PREC and traits instead of MAT and SWC on the 0.9 quantile.

Results of alternative models

When comparing the main model (using 0.9 as range shift, SD to describe niche breadth, and MAT and SWC to describe the thermal and moisture niche, respectively) to the three alternative models we found mostly similar results but also some differences.

For moths, the best alternative models were identical to the main model (Tables S2a, S3a, S4a, and S5a), i.e., the best model all contained mean thermal niche, thermal niche breadth, and wintering. The direction and relative size of the estimates as well as added information value of the variables were also the same (Table S6a).

For birds, the best alternative models 1 and 3 were identical to the main model (Tables S2b, S3b, S4b, and S5b), i.e., they all contained the same variables thermal niche breadth, wintering and number of brood. Alternative model 2 for birds, however, contained only thermal niche breadth (Table S3b). The direction of the variable estimates was the same in all models, but thermal niche breadth did not improve model fit in alternative model 2 (Table S6). In the main model and alternative model 1 and 3 for birds, the direction and relative size of the estimates as well ads the added information value for the variables were similar.

For butterflies, only alternative model 3 produced the same qualitative and similar quantitative result, with the moisture niche breadth having a positive effect on range shifts (Tables S2c, S3c, S4c, S5c, and S6c). In alternative models 2 and 3 mean moisture niche was the only variable selected for the best explaining model selected (Tables S3c and S5c) with a wetter niche indicating larger range shifts, but only significantly so for alternative model 2.

Model performance of alternative models

We assessed model performance (of the final model) by both visualizing and testing normality and heteroscedasticity of model residuals, collinearity (variance inflation factors), and influential outliers using the *performance* package in R (Lüdecke et al. 2021. We used these plots together with formal tests implemented in the performance package to evaluate model appropriateness.

For testing normality of residuals, we used the *check_normality* function in the *performance* package uses the Shapiro test where a p-value of 0.05 indicates deviation from normal distribution. For our alternative models 1 the normality test resulted in p-values below 0.001 for all species groups. Although the package developer notes that "this formal test almost always yields significant results for the distribution of residuals and visual inspection (e.g. Q-Q plots) are preferable", the visual inspection of residual normality (not shown) also indicate non-normality of residuals, wherefore we cannot trust that these models do violate the normality assumption of a linear model. Also, no transformation (log, cube, scale, Yeo-Johnson) of the dependent variables improved this metric. Therefore, we caution against definitive interpretations based on the alternative model 1. For alternative models 2 and 3 the normality test resulted in p-values of 0.040 and 0.043, 0.042 and 0.040, and 0.475 and 0.465 for the moth, bird, and butterfly alternative models 2 and 3, respectively. Since the residuals of the moth and bird models upon visual inspection were relatively well distributed along the qq-line and normal density distribution (not shown) and the p-values are close to 0.05, we consider the models not to violate the normality assumption of a linear model.

For heteroscedasticity tests, we employed the *check_ heteroscedasticity* function in the *performance* package which uses the Breush-Pagan test where a p-value < 0.05 indicates heteroscedasticity.
For alternative models 1, this test resulted in p-values of 0.112, 0.545, and <0.001 for the moth, bird, and butterfly models respectively. Therefore, we refitted the butterfly model using a linear regression that implements robust standard errors, namely the *lm_robust* function as implemented in the *estimatr* package (Blair et al. 2022). This approach adjusts standard errors to account for heteroscedasticity and is thus a more conservative approach in cases where the residuals show uneven patterns (Astivia & Zumbo 2019). The heteroscedasticity test resulted in p-values of 0.107 and 0.284, 0.089 and 0.397, and 0.893 and 0.667 for the moth, bird, and butterfly alternative models 2 and 3, respectively, indicating no heteroscedasticity in these models.

To test for collinearity among model variables we used the *check_collinearity* function in the *performance* package. The VIF values ranged between 1.05-1.10 for the moth alternative model 1. The bird and butterfly alternative models 1 contained only one variable, wherefore a collinearity test was not relevant. VIF values ranged between 1.10-1.13 and 1.07-1.71, 1.19-1.23 and 1.24-1.26 for the moth and bird alternative models 2 and 3, respectively. The butterfly alternative models 2 and 3 contained only one variable, wherefore a collinearity test was not relevant. Therefore, we can conclude that there were no multicollinearity issues in any of the alternative models.

We used the *check_outliers* as implemented in the *performance* package to check for influential outliers. No outliers were detected in the bird and moth models, but there was one outlier (case 41) detected in the alternative model 2 and 3 for butterflies.

Discussion on alternative models

The alternative models resulted in qualitatively similar results (see Text S5). Using change in the 0.75 quantile to measure range shifts (alt. model 1) resulted in differences for the bird (only thermal niche breadth had explanatory power, but not migratory behavior and number of broods) and butterfly model (mean moisture niche had explanatory power as opposed to moisture niche breadth). This indicates that, at

this scale of distribution shifts, the same mechanism cannot be identified to relate with range expansions or shifts as compared to the 0.9 quantile that better mirrors the leading range edge.

Standard deviation (a proxy for absolute niche breadth) and coefficient of variation (a proxy for relative niche breadth; alt. model 2) showed, overall, a similar effect on range shifts for all studies species groups, thus pointing to a lack of difference in the effect of relative versus absolute niche breadth at least at this scale.
Using other climatic metrics to describe the thermal and moisture niche (alt. model 3) indicated that the mean moisture niche had explanatory power as opposed to moisture niche breadth for butterflies but there was weak statistical support for this variable having any connection to range shifts overall.

References:

Astivia, Oscar L. Olvera and Zumbo, Bruno D. (2019) "Heteroskedasticity in Multiple Regression Analysis: What it is, How to Detect it and How to Solve it with Applications in R and SPSS," *Practical Assessment, Research, and Evaluation*: Vol. 24 , Article 1.

Blair, G., Cooper, J., Coppock, A., Humphreys, M., and Sonnet, L. (2022). estimatr: Fast Estimators for Design-Based Inference. R package version 1.0.0. https://CRAN.R-project.org/package=estimatr

Lüdecke et al., (2021). performance: An R Package for Assessment, Comparison and Testing of Statistical Models. Journal of Open Source Software, 6(60), 3139. https://doi.org/10.21105/joss.03139

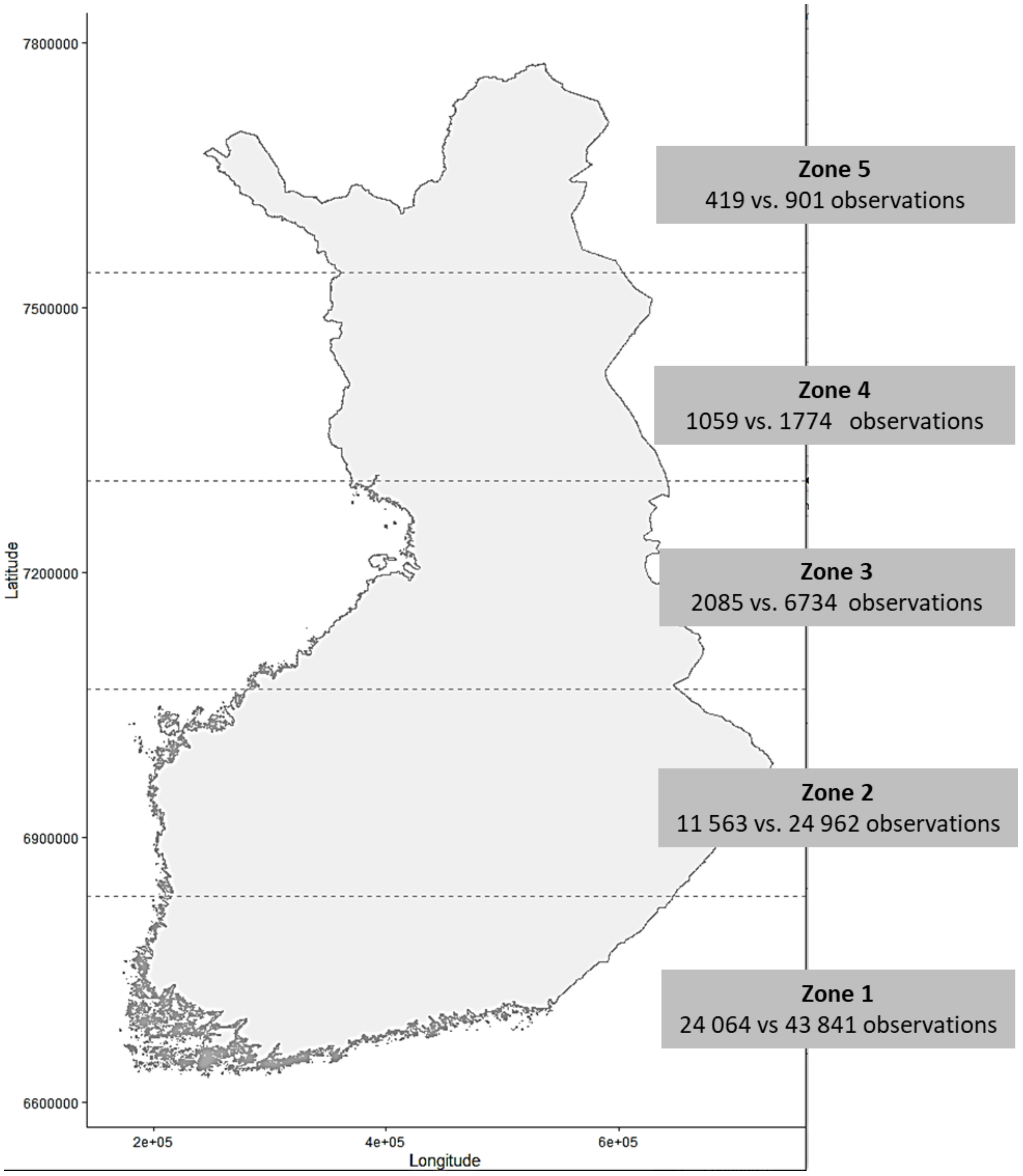

Figure S1. Latitudinal areas used for equalizing presence data on Lepidoptera within between the two periods. The total number of presence central distribution point squares for T_2 was substantially higher than for T1 due to overall increased sampling effort over time. To avoid effects caused by differences in overall observation intensity, we divided the data into these five latitudinal zones and randomly subsampled the pooled observations of all species at T_2 across the 304 Lepidoptera species that had at least 20 occupied grid cells per period, so that the total number of observations matched the number of observations at T_1 within each latitudinal zone. The number of observations at T_1 versus T_2 shown in the grey boxes alongside the map. Latitudes and longitudes in projection format ETRS-TM35FIN, EPSG:3067.

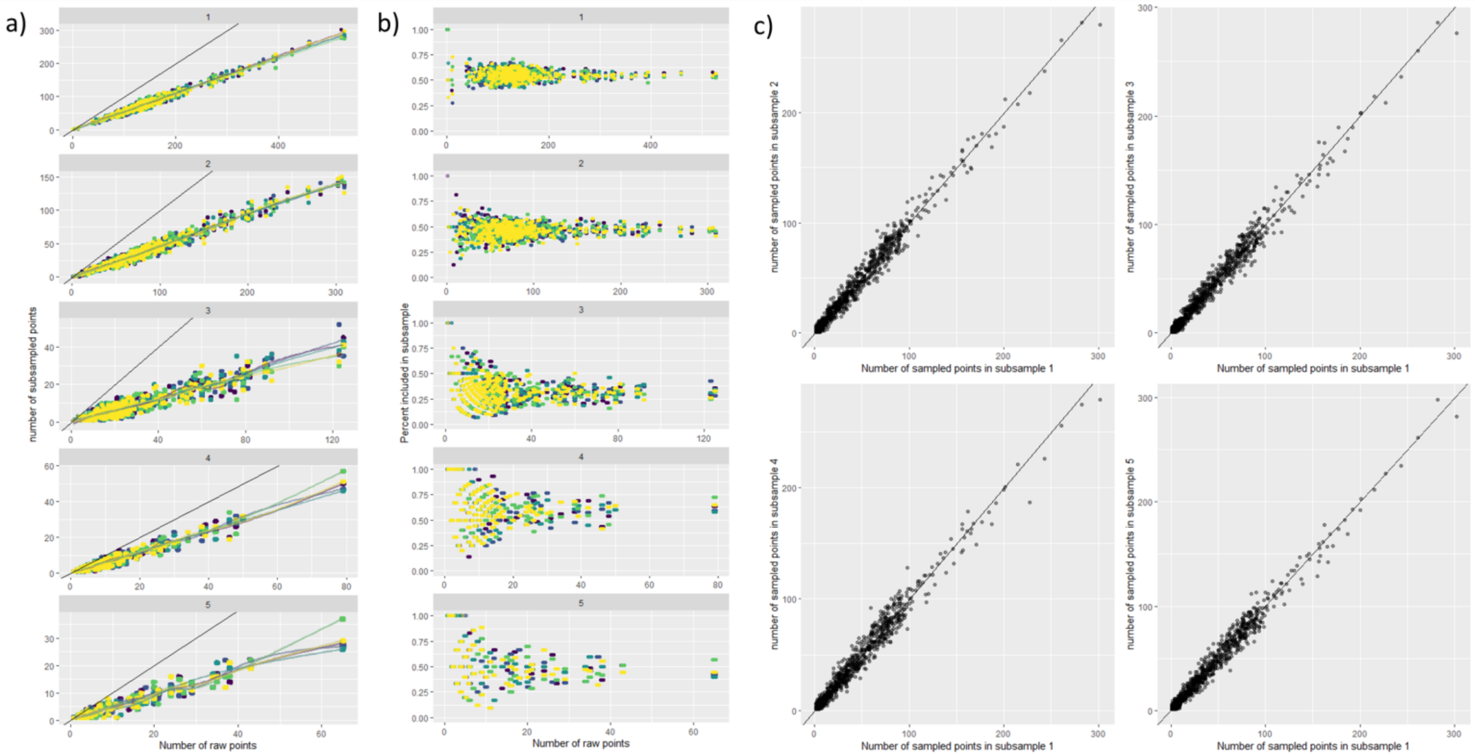

Figure S2. Number of distribution points of Lepidoptera included in the subsample for T_2 compared to the raw number of distribution points in T_2 shown for each latitudinal zone. a) Number of subsampled points compared to the number of points in the raw data with a fitted GAM line across each subsample. Each panel represents a latitudinal zone (Fig. S1). Different colors represent the five different subsamples and each point in a subsample represents one out of the 304 Lepidoptera species. b) Percent of points included in the subsampled compared to the number of points in the raw data. Horizontal jitter has been introduced to points in a) and b) to increase interpretability of overlapping values. c) Number of subsampled points in subsamples 2-5 compared to subsample 1 across all subsampling zones. Identity line (solid black line) added to all plots to ease interpretability. Overall, the absolute difference between raw and sampled data, as well as between samples, was greatest for species with many distribution points (a and c) and this difference was larger in zones where the total number of points was smaller (a). The percentage of included distribution points was most variable for species with low numbers of distribution points (b). Our approach to subsample within latitudinal zones removes variation caused by changes in effort that vary across space. Our approach using five-fold subsampling, analyzing each sample independently with quantitative regression and then averaging the estimates, reduces sampling-based stochasticity and imprecision and therefore results in a more conservative overall estimate.

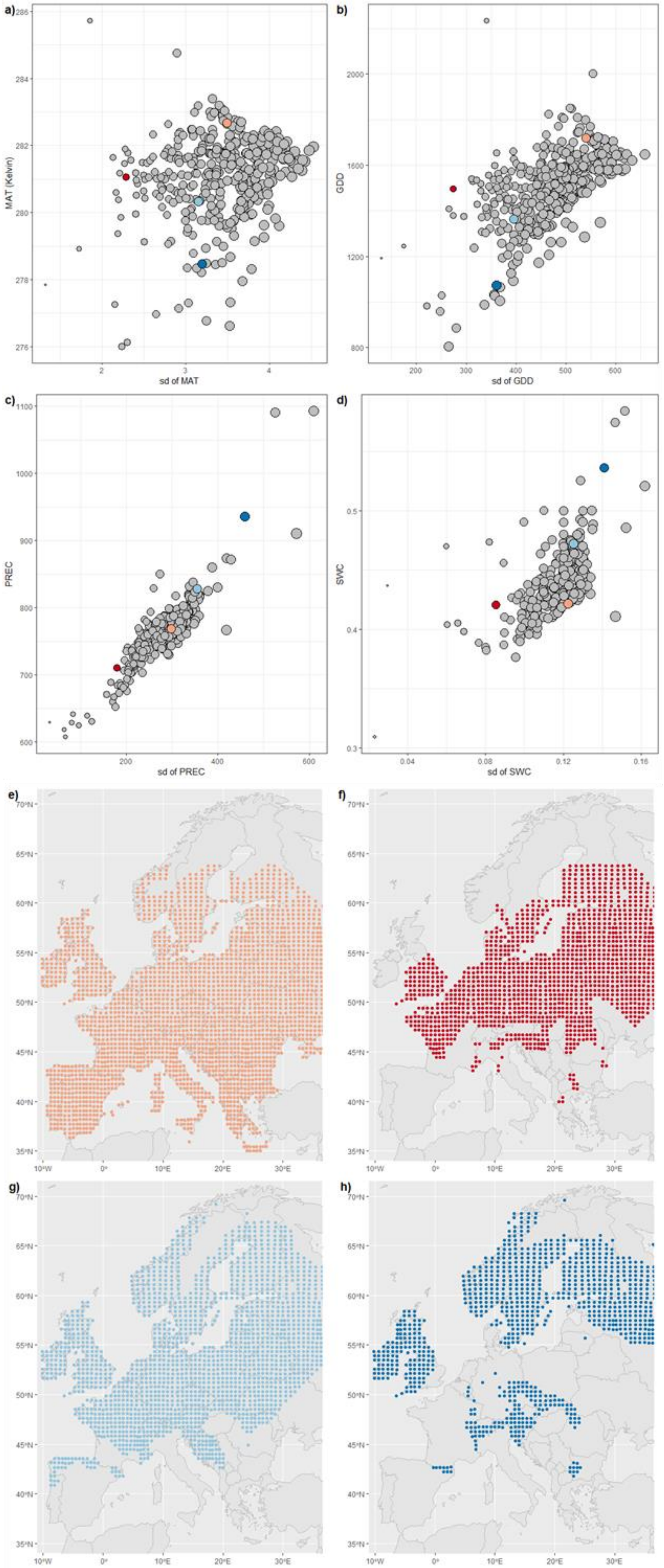

a)
MAT (Kelvin)
sd of MAT
b)
GDD
sd of GDD
c)
PREC
sd of PREC
d)
SWC
sd of SWC
e)
f)
g)
h)

Figure S3. Relationship between mean and standard deviation for climate niche metrics (a-d) and examples of data used to derive these (e-h). The metrics shown in panels (a-d) were used to calculate the coefficient of variation (cv), for a) mean annual temperature (MAT) b) growing degree days above 5°C between January-August (GDD), c) annual precipitation sum (PREC), d) soil water content (SWC). Circle size gives CV for each species. Colored points show the metric values of the four example species in (e-h) on a backdrop of all species in this study. Panels (e-h) show examples atlas data used to calculate the climatic niche metrics moth species: e) *Gymnoscelis rufifasciata* with a large range and relatively southern 0.9 quantile at T_1, f) *Idaea emarginata* with a small range and relatively southern 0.9 quantile at T_1 g) *Plemyria rubiginata* with a large range and relatively northern 0.9 quantile at T_1, and h) *Venusia cambrica* with a small range and relatively northern 0.9 quantile at T_1. The atlas data for these moth species are published in Heidrich et al. (2018). Climatic niche metrics and range size were derived from atlas data (Text S3) while the position of the 0.9 quantile in T_1 was estimated based on distribution data (Texts S1 and S2).

Reference:

Heidrich, L., Friess, N., Fiedler, K., Brändle, M., Hausmann, A., Brandl, R., *et al.* (2018) The dark side of Lepidoptera: Colour lightness of geometrid moths decreases with increasing latitude. *Global Ecology and Biogeography*, **27**, 407–416.

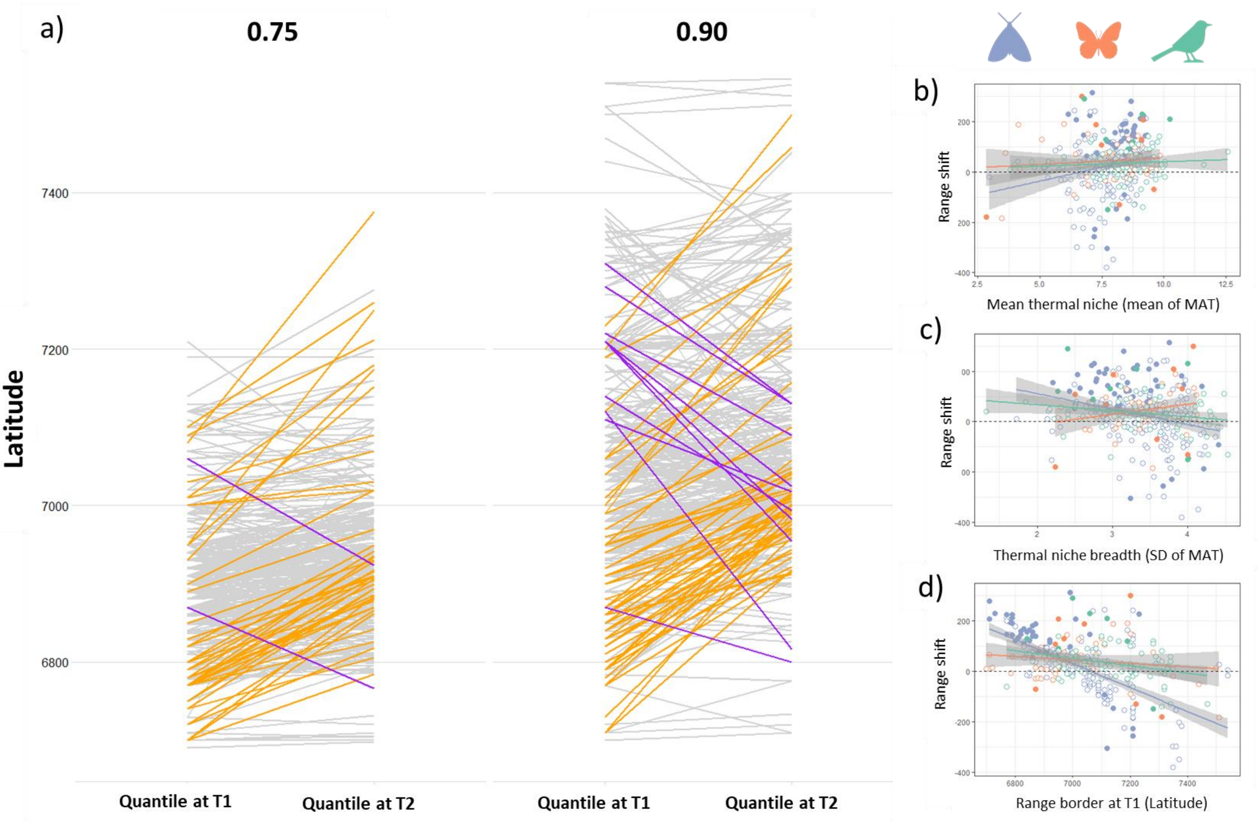

Figure S4. Shift in range based on metrics describing the range position and extent of species. a) 0.75 and 0.9 quantiles of the distribution along latitudinal gradient and across taxonomic groups. N= 383 species (Moths= 239; Butterflies= 57; Birds= 87). Grey lines = CI for the estimated difference in quantile between T_1 and T_2 includes zero; orange and purple CI for a positive (yellow) or negative (purple) estimated difference in quantile between T_1 and T_2 does not include 0. Latitudes in projection KKJ / Finland Uniform Coordinate System, EPSG:2393. Univariate plots for the effect of b) mean thermal niche, c) breadth of thermal niche, and d) latitudinal position of range edge at T_1 on shift in 0.9 quantile between T_1 and T_2 (range shift; latitudes in projection KKJ / Finland Uniform Coordinate System, EPSG:2393. The fitted lines in b)-d) are linear regressions based on a univariate linear models and points are raw data. MAT= Mean annual temperature. Model comparison of univariate models b)-d) using AIC for moths indicated that latitudinal range edge position at T_1 (AIC= 6239.54) explained the data better then mean thermal niche (AIC=6380.90) or thermal niche breadth (AIC=6375.11). For birds, thermal niche breadth (AIC= 977.59) and latitudinal range edge position at T_1 (978.15) had similar explanatory power and both explained the data slightly better than mean thermal niche (AIC =981.42). For butterflies, the thermal niche breadth (AIC = 3448.89) explained the data better than did mean thermal niche (AIC = 3450.64) or latitudinal position at T_1 (AIC = 3451.27).

Thus, we see some indications that species with a lower northern range edge in T1 tended to move further north. Rapoport's rule (Stevens, 1989) states that species at higher latitudes have tend to have broader ranges (and thus potentially broader niches) than species occurring closer to the equator. Yet, among our study species, those species with warmer thermal niches tended to also have broader niches (Fig. S3). At first glance, our data thus seems not to support Rapoport's rule. However, this effect is likely caused by the

delimitation of our study area. Namely, we only examined species occurring in Finland but measured their climatic niches from across the whole of Europe. Because of this, the “warmer” the species is, the larger its range needs to be within Europe in order to both occur within the borders of Finland and to occupy warmer conditions. Therefore, the niche breadth, which would be narrower for species at lower (warmer) latitudes, is potentially a more proximate explanation for why we observe warmer species moving to higher latitudes at higher rates than species inhabiting colder areas.

Reference:

Stevens, G.C. (1989) The Latitudinal Gradient in Geographical Range: How so Many Species Coexist in the Tropics. *The American Naturalist*, 133.

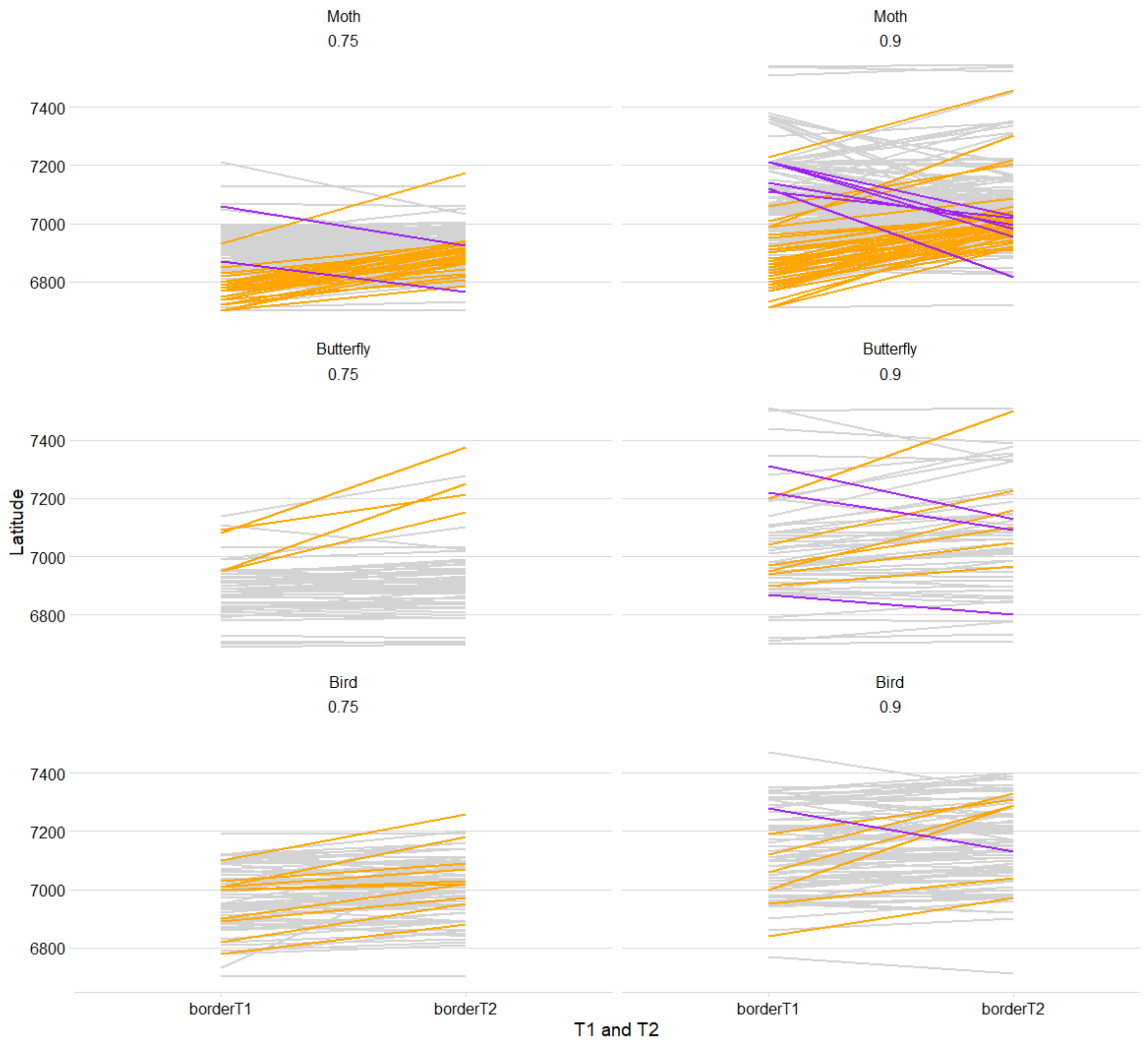

Figure S5. Shift in 0.75 (left-hand side) and 0.9 quantiles (right-hand side) of the distribution along the latitudinal gradient per taxonomic group. Birds= 87; Butterflies= 57; Moths= 239. Grey lines = CI for the estimated difference in quantile between T_1 and T_2 includes zero; orange and purple CI for a positive (yellow) or negative (purple) estimated difference in quantile between T_1 and T_2 does not include 0. Latitude in projection projection KKJ / Finland Uniform Coordinate System, EPSG:2393.

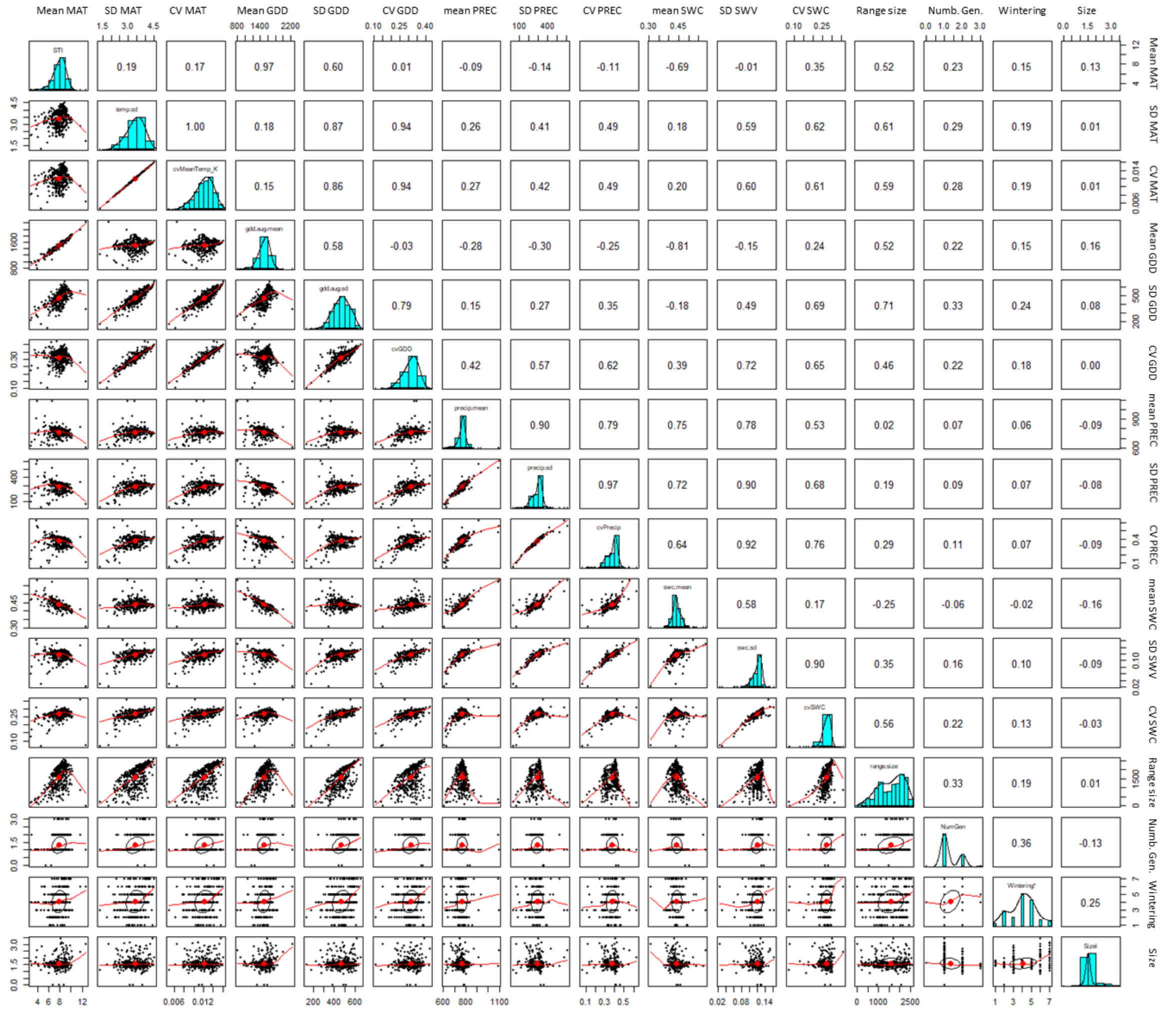

Figure S6. Correlation matrix of all explanatory variables. Created using the *pairs.panel* function in the *psych* package (Revelle 2022). MAT= Mean annual temperature; GDD= Growing degree days above 5 until august; PREC= annual precipitation sum; SWC= Soil water content; SD= Standard deviation; CV= Coefficient of variation.

Reference:

Revelle, W. (2022) psych: Procedures for Personality and Psychological Research, Northwestern University, Evanston, Illinois, USA, Version = 2.2.5.

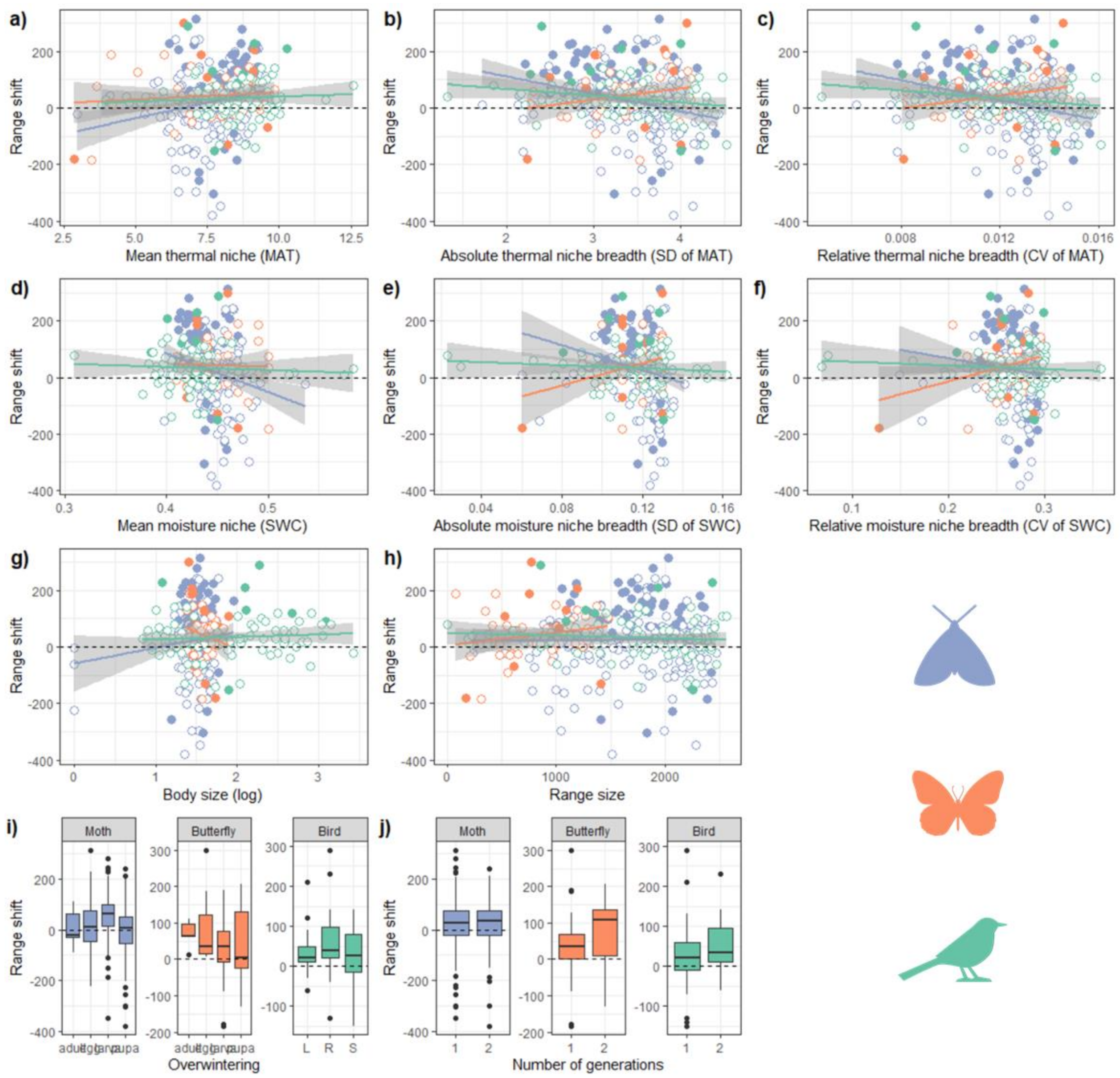

Fig. S7. Univariate plots with for all explanatory variables included in the initial model, from which the best explaining variables were selected using model selection. In a)-h) fitted lines are linear regressions based on univariate linear models and points are raw data. MAT= Mean annual temperature; SWC= Soil water content; SD= Standard deviation; CV= Coefficient of variation.

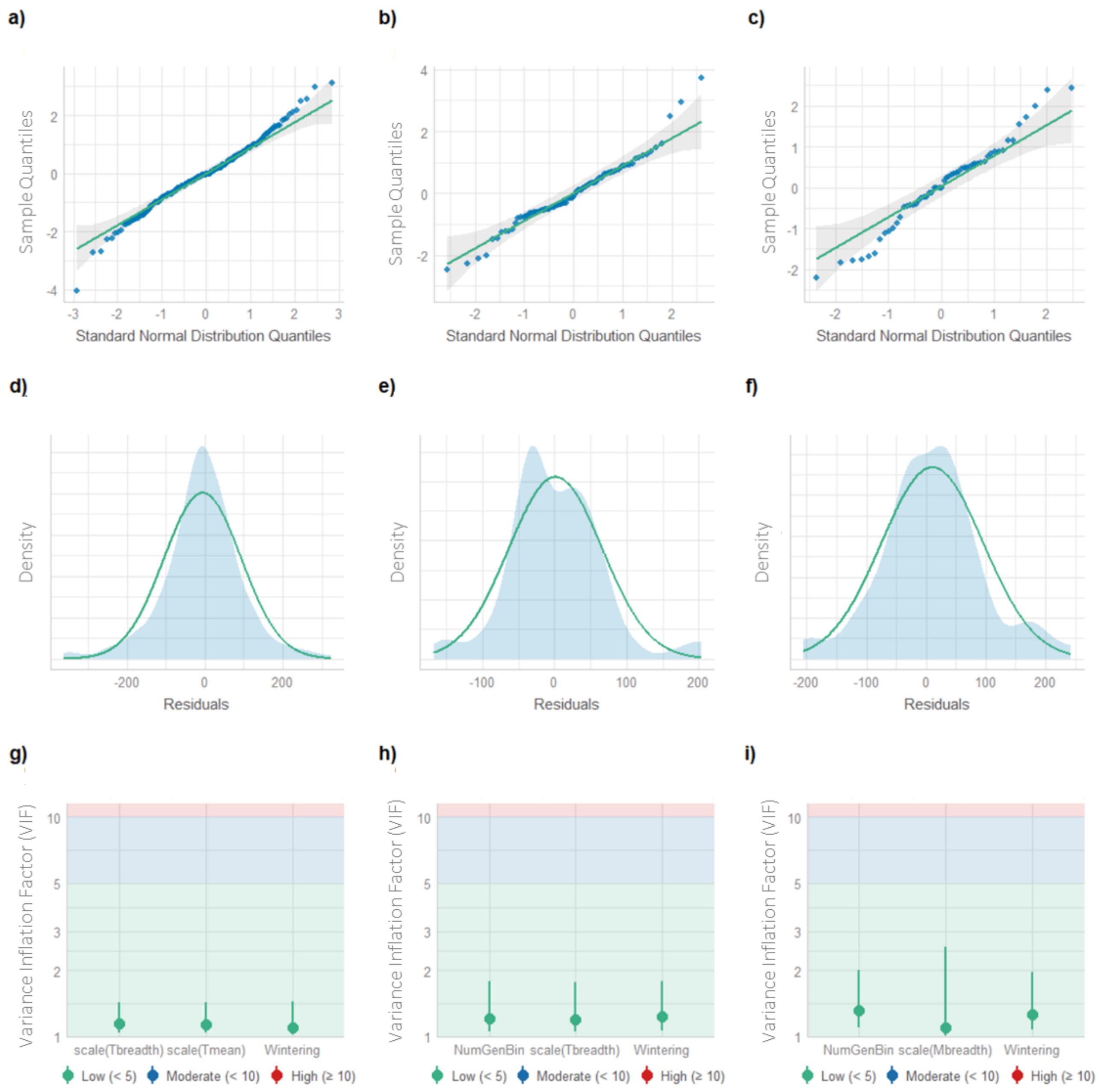

Figure S8. Model performance of main final models (presented in the main text). We assessed model performance (of the final model) by both visualizing and testing normality and heteroscedasticity of model residuals, collinearity (variance inflation factors), and influential outliers using the *performance* package in R (Lüdecke et al. 2021. Figure S8 show residual normality plots (a-f) and variance inflation plots (g-i) for the main models for moths (a, d, and g), birds (b, e, and h), and butterflies (c, f, and i). We used these together with formal tests implemented in the *performance* package to evaluate model appropriateness. For testing normality of residuals the *check_normality* function in the *performance* package uses the Shapiro test where a p-value of 0.05 indicates deviation from normal distribution. For our main models the test resulted in p-values of 0.040, 0.043, and 0.430 for the moth, bird, and butterfly model, respectively. The package developer, however, note that "this formal test almost always yields significant results for the distribution of residuals and visual inspection (e.g. Q-Q plots) are preferable". Since the residuals of the moth and bird models upon visual inspection are relatively well distributed along the qq-line and normal density distribution and the p-values are close to 0.05, we consider the models not to violate the normality assumption of a linear model. Also, no transformation (log, cube, scale, Yeo-Johnson) of the dependent

variables improved this metric. For heteroscedasticity tests, we employed the *check_ heteroscedasticity* function in the *performance* package which uses the Breush-Pagan test where a p-value < 0.05 indicates heteroscedasticity. For our main full models, this test resulted in p-values of 0.107, 0.104, and 0.964 for the moth, bird, and butterfly models respectively. To test for collinearity among model variables we used the *check_collinearity* function in the *performance* package. The VIF values ranged between 1.10-1.14, and 1.19-1.23 for the moth and bird models, respectively. The butterfly model contained only one variable, wherefore a collinearity test was not relevant. Therefore, we can conclude that there were no multicollinearity issues in our full models. We used the *check_outliers* as implemented in the *performance* package to check for influential outliers and it revealed that no outliers were detected in the moth or bird models, but one outlier (case 41) was identified as an outlier with a cook value of 0.7 in the butterfly model.

References:

Astivia, Oscar L. Olvera and Zumbo, Bruno D. (2019) "Heteroskedasticity in Multiple Regression Analysis: What it is, How to Detect it and How to Solve it with Applications in R and SPSS," *Practical Assessment, Research, and Evaluation*: Vol. 24 , Article 1.

Blair, G., Cooper, J., Coppock, A., Humphreys, M., and Sonnet, L. (2022). estimatr: Fast Estimators for Design-Based Inference. R package version 1.0.0. https://CRAN.R-project.org/package=estimatr

Table S1. Number of species per categorical trait group.

**Generations/broods per year**		
	On or less	Two or more
Moth	166	73
Butterfly	45	12
Bird	57	30

**Overwintering mode**							
	Adult	Egg	Larva	Pupa	Resident	Short-dist. migr.	Long-dist. migr.
Moth	9	53	85	92	NA	NA	NA
Butterfly	5	7	37	8	NA	NA	NA
Bird	NA	NA	NA	NA	26	24	37

Table S2. Results of the best subsets regression of the main model comparing all possible combinations of our eight explanatory variables ($2^8 = 256$ potential models). This method provides a list of the best fitting models for models with one, two, three …8 variables, based on a balance of several criteria like R^2 and AIC. The produced list of best fitting models includes a diverse range of such model fit metrics for the user to use for further evaluations. AIC: Akaike Information Criteria; SBC: Schwarz Bayesian Criteria; MSEP: Estimated error of prediction, assuming normality; FPE: Final Prediction Error; HSP: Hocking's Sp; APC: Amemiya Prediction Criteria. From this list we chose the best explaining model based on the lowest AIC value (Akaike's Information Criterionwhile also considering increase in adjusted and predicted R^2. However, in order to choose a less parsimonious model (a model with more variables) the AIC needed to be at least 2 units lower since models with AIC values differing with less than 2 units can be considered to have the same information value. With this we sought to identify a minimal model that fits the data best and thereby identify the variables that help explain observed range shifts.

a) Best Subsets Regression for Moths

Predictors	Predictors	Adj. R-Square	Pred. R-Square	R-Square	AIC	SBC	MSEP	FPE	HSP	APC
Tbreadth	1	0.0744	0.0705	0.0587	2911.2071	2921.6365	2683596.3200	11322.3949	47.5781	0.9412
Tmean + Tbreadth	2	0.1438	0.1365	0.1227	2894.5814	2908.4873	2492964.4688	10561.5516	44.3856	0.8780
**Tmean + Tbreadth + Wintering**	**3**	**0.1872**	**0.1697**	**0.1491**	**2888.1533**	**2912.4885**	**2376755.3433**	**10197.4387**	**42.8615**	**0.8405**
Tmean + Tbreadth + Wintering + Numb. Gen.	4	0.1954	0.1746	0.1505	2887.7337	2915.5454	2362913.2682	10179.9573	42.7955	0.8390
Tmean + Tbreadth + Wintering + Numb. Gen. + Size	5	0.2034	0.1792	0.1521	2887.3376	2918.6257	2349424.8509	10163.5180	42.7354	0.8376
Tmean + Tbreadth + Wintering + Numb. Gen. + Size + Range size	6	0.2054	0.1778	0.1463	2888.7340	2923.4987	2353644.4771	10223.5218	42.9983	0.8426
Tmean + Tbreadth + Mmean + Wintering + Numb. Gen. + Size + Range size	7	0.2066	0.1754	0.1403	2890.3699	2928.6110	2360279.0589	10294.2112	43.3078	0.8483
Tmean + Tbreadth + Mmean + Mbreadth + Wintering + Numb. Gen. + Size + Range size	8	0.2072	0.1725	0.1333	2892.1833	2933.9008	2368735.5377	10373.1177	43.6536	0.8548

b) Best Subsets Regression for Birds

Predictors	Predictors	Adj. R-Square	Pred. R-Square	R-Square	AIC	SBC	MSEP	FPE	HSP	APC
Wintering	1	0.0547	0.0321	-0.02	992.2917	1002.1553	427193.1068	5082.9126	59.1511	0.9898
Tbreadth + Wintering	2	0.1230	0.0913	0.0272	987.7673	1000.0968	401103.4454	4826.1110	56.2077	0.9397
**Tbreadth + Wintering + Numb. Gen.**	**3**	**0.1877**	**0.1480**	**0.0759**	**983.1005**	**997.8960**	**376046.0838**	**4574.8922**	**53.3390**	**0.8906**
Tbreadth + Wintering + Numb. Gen. + Size	4	0.1998	0.1505	0.0304	983.7848	1001.0461	374974.4672	4611.9854	53.8436	0.8977
Tbreadth + Wintering + Numb. Gen. Size + Range size	5	0.2153	0.1564	0.0393	984.0905	1003.8177	372339.3226	4629.3525	54.1336	0.9010
Tbreadth + Mmean + Wintering + Numb. Gen. + Size + Range size	6	0.2189	0.1497	0.0302	985.6845	1007.8777	375297.0917	4716.3003	55.2543	0.9178
Tmean + Tbreadth + Mmean + Wintering + Numb. Gen. + Size + Range size	7	0.2238	0.1442	0.014	987.1365	1011.7956	377722.0851	4797.2725	56.3243	0.9334
Tmean + Tbreadth + Mmean + Mbreadth + Wintering + Numb. Gen. + Size + Range size	8	0.2249	0.1343	-0.0129	989.0148	1016.1398	382092.7777	4903.8645	57.7159	0.9539

c) Best Subsets Regression for Butterflies

Predictors	Predictors	Adj. R-Square	Pred. R-Square	R-Square	AIC	SBC	MSEP	FPE	HSP	APC
**Mbreadth**	**1**	**0.0751**	**0.0583**	**-0.0334**	**673.4953**	**679.6244**	**421408.7881**	**7652.3705**	**136.9068**	**0.9921**
Tbreadth + Wintering	2	0.1446	0.0788	-0.0531	675.0447	687.3031	397111.0430	7612.8112	136.4560	0.9505
Mbreadth + Wintering + Numb. Gen.	3	0.1772	0.0965	-0.0708	674.8316	689.1329	389333.5937	7590.9162	136.4068	0.9470
Tbreadth + Mbreadth + Wintering + Numb. Gen.	4	0.1920	0.0951	-0.0912	675.7916	692.1360	389790.5296	7727.3834	139.2982	0.9633
Tbreadth + Mbreadth + Wintering + Numb. Gen. + Range size	5	0.1947	0.0797	-0.1331	677.6021	695.9896	396266.7385	7985.6710	144.5026	0.9948
Tmean + Tbreadth + Mmean + Mbreadth + Wintering + Numb. Gen.	6	0.2044	0.0718	-0.197	678.9105	699.3410	399477.1260	8181.5587	148.7082	1.0183
Tmean + Tbreadth + Mmean + Mbreadth + Wintering + Numb. Gen. + Range size	7	0.2056	0.0534	-0.253	680.8292	703.3028	407218.7352	8474.1043	154.8154	1.0538
Tmean + Tbreadth + Mmean + Mbreadth + Wintering + Numb. Gen. + Size + Range size	8	0.2057	0.0330	-0.2993	682.8228	707.3394	415835.6773	8790.5293	161.5281	1.0922

Table S3. Results of the best subsets regression of the alternative model 1 comparing all possible combinations of our eight explanatory variables (2^8 = 256 potential models). This method provides a list of the best fitting models for models with one, two, three …8 variables, based on a balance of several criteria like R^2 and AIC. The produced list of best fitting models includes a diverse range of such model fit metrics for the user to use for further evaluations. AIC: Akaike Information Criteria; SBC: Schwarz Bayesian Criteria; MSEP: Estimated error of prediction, assuming normality; FPE: Final Prediction Error; HSP: Hocking's Sp; APC: Amemiya Prediction Criteria. From this list we chose the best explaining model based on the lowest AIC value (Akaike's Information Criterionwhile also considering increase in adjusted and predicted R^2. However, in order to choose a less parsimonious model (a model with more variables) the AIC needed to be at least 2 units lower since models with AIC values differing with less than 2 units can be considered to have the same information value. With this we sought to identify a minimal model that fits the data best and thereby identify the variables that help explain observed range shifts.

Alternative model 1 where we used the 0.75 quantile of distribution points to measure shift in northern range edge

a) Best Subsets Regression for Moths

Predictors	Predictors	Adj. R-Square	Pred. R-Square	R-Square	AIC	SBC	MSEP	FPE	HSP	APC
Tbreadth + Wintering	2	0.1816	0.1676	0.1479	2558.7755	2579.6343	601511.4400	2570.1098	10.8011	0.8393
**Tmean + Tbreadth + Wintering**	**3**	**0.2268**	**0.2102**	**0.1874**	**2547.1712**	**2571.5065**	**570656.9996**	**2448.3966**	**10.2910**	**0.7995**
Tmean + Tbreadth + Wintering + Numb. Gen.	4	0.2301	0.2102	0.1847	2548.1625	2575.9742	570692.4943	2458.6705	10.3360	0.8028
Tmean + Tbreadth + Wintering + Numb. Gen. + Size	5	0.2329	0.2097	0.178	2549.2785	2580.5667	571036.2772	2470.2801	10.3870	0.8066
Tmean + Tbreadth + Mbreadth + Wintering + Numb. Gen. + Size	6	0.2341	0.2074	0.1709	2550.9242	2585.6888	572658.7659	2487.4570	10.4618	0.8121
Tmean + Tbreadth + Mmean + Mbreadth + Wintering + Numb. Gen. + Size	7	0.2343	0.2042	0.1651	2552.8589	2591.1000	574991.4766	2507.7898	10.5503	0.8188
Tmean + Tbreadth + Mmean + Mbreadth + Wintering + Numb. Gen. + Size + Range size	8	0.2345	0.2009	0.1571	2554.8044	2596.5220	577370.7773	2528.4102	10.6404	0.8255

b) Best Subsets Regression for Birds

Predictors	Predictors	Adj. R-Square	Pred. R-Square	R-Square	AIC	SBC	MSEP	FPE	HSP	APC
**Tbreadth**	**1**	**0.0265**	**0.0150**	**-0.0152**	**961.5248**	**968.9225**	**306918.6079**	**3608.8762**	**41.9974**	**1.0193**
Tmean + Range size	2	0.0627	0.0404	-0.0297	960.2297	970.0933	299071.8126	3555.6189	41.4108	1.0043
Tmean + Tbreadth + Wintering	3	0.0825	0.0378	-0.0348	962.3697	977.1651	296315.7685	3604.9111	42.0299	1.0059
Tmean + Wintering + Numb. Gen. + Range size	4	0.0957	0.0399	-0.0799	963.1053	980.3667	295646.1165	3636.2891	42.4527	1.0145
Tmean + Tbreadth + Wintering + Numb. Gen. + Size	5	0.1069	0.0400	-0.0777	964.0217	983.7490	295636.3315	3675.6924	42.9819	1.0254
Tmean + Tbreadth + Wintering + Numb. Gen. + Size + Range size	6	0.1109	0.0321	-0.1374	965.6351	987.8283	298051.3267	3745.5648	43.8815	1.0447
Tmean + Tbreadth + Mbreadth + Wintering + Numb. Gen. + Size + Range size	7	0.1112	0.0200	-0.1752	967.6054	992.2644	301769.1980	3832.6302	44.9985	1.0688
Tmean + Tbreadth + Mmean + Mbreadth + Wintering + Numb. Gen. + Size + Range size	8	0.1112	0.0073	-0.1898	969.6043	996.7293	305684.6101	3923.2249	46.1743	1.0939

c) Best Subsets Regression for Butterflies

Predictors	Predictors	Adj. R-Square	Pred. R-Square	R-Square	AIC	SBC	MSEP	FPE	HSP	APC
**Mmean**	**1**	**0.1520**	**0.1366**	**0.0629**	**639.8470**	**645.9761**	**233523.2763**	**4240.5538**	**75.8668**	**0.9097**
Mmean + Wintering	2	0.1971	0.1353	-0.002	642.7316	654.9899	225274.3383	4318.6183	77.4092	0.8921
Tbreadth + Mmean + Wintering	3	0.2459	0.1720	-0.0069	641.1577	655.4590	215652.0146	4204.6112	75.5557	0.8679
Tmean + Tbreadth + Mbreadth + Wintering	4	0.2700	0.1824	-0.0767	641.3053	657.6497	212849.8643	4219.6318	76.0655	0.8704
Tmean + Tbreadth + Mbreadth + Wintering + Numb. Gen.	5	0.2860	0.1840	-0.0664	642.0455	660.4330	212360.9682	4279.5538	77.4395	0.8820
Tmean + Tbreadth + Mbreadth + Wintering + Numb. Gen. + Size	6	0.2877	0.1690	-0.0869	643.9068	664.3374	216168.3830	4427.2730	80.4702	0.9117
Tmean + Tbreadth + Mbreadth + Wintering + Numb. Gen. + Size + Range size	7	0.2896	0.1536	-0.134	645.7542	668.2277	220081.5637	4579.8338	83.6700	0.9424
Tmean + Tbreadth + Mmean + Mbreadth + Wintering + Numb. Gen. + Size + Range size	8	0.2896	0.1352	-0.1657	647.7538	672.2704	224762.6585	4751.3545	87.3073	0.9768

Table S4. Results of the best subsets regression of the alternative model 2 comparing all possible combinations of our eight explanatory variables (2^8 = 256 potential models). This method provides a list of the best fitting models for models with one, two, three …8 variables, based on a balance of several criteria like R^2 and AIC. The produced list of best fitting models includes a diverse range of such model fit metrics for the user to use for further evaluations. AIC: Akaike Information Criteria; SBC: Schwarz Bayesian Criteria; MSEP: Estimated error of prediction, assuming normality; FPE: Final Prediction Error; HSP: Hocking's Sp; APC: Amemiya Prediction Criteria. From this list we chose the best explaining model based on the lowest AIC value (Akaike's Information Criterionwhile also considering increase in adjusted and predicted R^2. However, in order to choose a less parsimonious model (a model with more variables) the AIC needed to be at least 2 units lower since models with AIC values differing with less than 2 units can be considered to have the same information value. With this we sought to identify a minimal model that fits the data best and thereby identify the variables that help explain observed range shifts. Tmean = mean thermal niche; Tbreadth = breadth of thermal niche; Mmean = mean moisture niche; Mbreadth = breadth of moisture niche; Wintering = overwintering mode; Numb. Gen. = number of generations or broods per season; Size = body size; Range size = range size across Europe.

Alternative model 2 where we used the CV (realtive niche breadth) instead of SD (absolute niche breadth) to measure niche breadth

a) Best Subsets Regression for Moths

Predictors	Predictors	Adj. R-Square	Pred. R-Square	R-Square	AIC	SBC	MSEP	FPE	HSP	APC
Tbreadth	1	0.0774	0.0736	0.0617	2910.4179	2920.8473	2674750.0186	11285.0714	47.4213	0.9381
Tbreadth + Range size	2	0.1441	0.1368	0.1225	2894.5003	2908.4061	2492117.9724	10557.9654	44.3706	0.8777
**Tmean + Tbreadth + Wintering**	**3**	**0.1869**	**0.1695**	**0.1488**	**2888.2233**	**2912.5585**	**2377451.4794**	**10200.4254**	**42.8740**	**0.8407**
Tmean + Tbreadth + Wintering + Numb. Gen.	4	0.1951	0.1743	0.1502	2887.8186	2915.6303	2363752.5699	10183.5732	42.8107	0.8393
Tmean + Tbreadth + Wintering + Numb. Gen. + Size	5	0.2030	0.1789	0.1517	2887.4401	2918.7283	2350433.5103	10167.8814	42.7538	0.8380
Tmean + Tbreadth + Wintering + Numb. Gen. + Size + Range size	6	0.2050	0.1773	0.1458	2888.8555	2923.6201	2354840.8808	10228.7186	43.0202	0.8430
Tmean + Tbreadth + Mmean + Wintering + Numb. Gen. + Size + Range size	7	0.2062	0.1750	0.1398	2890.4879	2928.7290	2361444.6549	10299.2948	43.3292	0.8488
Tmean + Tbreadth + Mmean + Mbreadth + Wintering + Numb. Gen. + Size + Range size	8	0.2065	0.1717	0.1323	2892.4097	2934.1273	2370980.5698	10382.9491	43.6950	0.8556

b) Best Subsets Regression for Birds

Predictors	Predictors	Adj. R-Square	Pred. R-Square	R-Square	AIC	SBC	MSEP	FPE	HSP	APC
Wintering	1	0.0547	0.0321	-0.02	992.2917	1002.1553	427193.1068	5082.9126	59.1511	0.9898
Tbreadth + Wintering	2	0.1251	0.0935	0.0295	987.5557	999.8853	400129.3027	4814.3900	56.0712	0.9374
**Tbreadth + Wintering + Numb. Gen.**	**3**	**0.1894**	**0.1499**	**0.0778**	**982.9128**	**997.7083**	**375235.5698**	**4565.0317**	**53.2240**	**0.8887**
Tbreadth + Wintering + Numb. Gen. + Size	4	0.2014	0.1521	0.0327	983.6112	1000.8726	374227.1851	4602.7943	53.7363	0.8959
Tbreadth + Wintering + Numb. Gen. + Size + Range size	5	0.2158	0.1570	0.0403	984.0275	1003.7547	372069.7846	4626.0013	54.0944	0.9003
Tbreadth + Mmean + Wintering + Numb. Gen. + Size + Range size	6	0.2204	0.1513	0.0321	985.5211	1007.7142	374592.8564	4707.4503	55.1506	0.9160
Tmean + Tbreadth + Mmean + Wintering + Numb. Gen. + Size + Range size	7	0.2243	0.1447	0.0145	987.0846	1011.7437	377496.7176	4794.4102	56.2906	0.9328
Tmean + Tbreadth + Mmean + Mbreadth + Wintering + Numb. Gen. + Size + Range size	8	0.2250	0.1344	-0.0176	989.0058	1016.1308	382053.1045	4903.3554	57.7099	0.9538

c) Best Subsets Regression for Butterflies

Predictors	Predictors	Adj. R-Square	Pred. R-Square	R-Square	AIC	SBC	MSEP	FPE	HSP	APC
**Mbreadth**	**1**	**0.0778**	**0.0611**	**-0.0495**	**673.3287**	**679.4578**	**420178.9789**	**7630.0383**	**136.5073**	**0.9892**
Tbreadth + Wintering	2	0.1434	0.0775	-0.0546	675.1220	687.3803	397649.7243	7623.1380	136.6412	0.9517
Tbreadth + Mbreadth + Wintering	3	0.1709	0.0896	-0.1008	675.2631	689.5645	392292.5383	7648.6074	137.4434	0.9542
Tbreadth + Mbreadth + Wintering + Numb. Gen.	4	0.1890	0.0917	-0.1353	676.0075	692.3519	391269.9223	7756.7116	139.8269	0.9670
Tbreadth + Mbreadth + Wintering + Numb. Gen. + Range size	5	0.1968	0.0821	-0.1561	677.4534	695.8409	395234.3813	7964.8666	144.1262	0.9922
Tmean + Tbreadth + Mmean + Mbreadth + Wintering + Numb. Gen.	6	0.2003	0.0670	-0.2023	679.2083	699.6388	401569.6323	8224.4146	149.4871	1.0237
Tmean + Tbreadth + Mmean + Mbreadth + Wintering + Numb. Gen. + Range size	7	0.2016	0.0487	-0.258	681.1153	703.5888	409267.3569	8516.7355	155.5942	1.0591
Tmean + Tbreadth + Mmean + Mbreadth + Wintering + Numb. Gen. + Size + Range size	8	0.2017	0.0281	-0.3043	683.1080	707.6246	417922.0478	8834.6340	162.3385	1.0977

Table S5. Results of the best subsets regression of the alternative model 3 comparing all possible combinations of our eight explanatory variables (2^8 = 256 potential models). This method provides a list of the best fitting models for models with one, two, three …8 variables, based on a balance of several criteria like R^2 and AIC. The produced list of best fitting models includes a diverse range of such model fit metrics for the user to use for further evaluations. AIC: Akaike Information Criteria; SBC: Schwarz Bayesian Criteria; MSEP: Estimated error of prediction, assuming normality; FPE: Final Prediction Error; HSP: Hocking's Sp; APC: Amemiya Prediction Criteria. From this list we chose the best explaining model based on the lowest AIC value (Akaike's Information Criterionwhile also considering increase in adjusted and predicted R^2. However, in order to choose a less parsimonious model (a model with more variables) the AIC needed to be at least 2 units lower since models with AIC values differing with less than 2 units can be considered to have the same information value. With this we sought to identify a minimal model that fits the data best and thereby identify the variables that help explain observed range shifts. Tmean = mean thermal niche; Tbreadth = breadth of thermal niche; Mmean = mean moisture niche; Mbreadth = breadth of moisture niche; Wintering = overwintering mode; Numb. Gen. = number of generations or broods per season; Size = body size; Range size = range size across Europe.

Alternative model 3 where we used the GDD instead of MAT to describe the thermal niche and PREC instead of SWC to describe the moisture niche.

a) Best Subsets Regression for Moths

Predictors	Predictors	Adj. R-Square	Pred. R-Square	R-Square	AIC	SBC	MSEP	FPE	HSP	APC
Wintering	1	0.0671	0.0551	0.0388	2917.0950	2934.4773	2704878.5625	11509.3121	48.3636	0.9487
Tmean + Tbreadth	2	0.1255	0.1181	0.1039	2899.6267	2913.5325	2546149.9310	10786.8741	45.3326	0.8967
**Tmean + Tbreadth + Wintering**	**3**	**0.1783**	**0.1607**	**0.1397**	**2890.7465**	**2915.0818**	**2402684.5028**	**10308.6874**	**43.3290**	**0.8497**
Tmean + Tbreadth + Wintering + Numb. Gen.	4	0.1832	0.1621	0.1376	2891.3095	2919.1212	2398531.0005	10333.4064	43.4406	0.8517
Tmean + Tbreadth + Wintering + Numb. Gen. + Size	5	0.1891	0.1646	0.137	2891.5791	2922.8673	2391492.8050	10345.5023	43.5006	0.8526
Tmean + Tbreadth + Mmean + Wintering + Numb. Gen. + Size	6	0.1949	0.1669	0.1379	2891.8753	2926.6399	2384783.5502	10358.7805	43.5672	0.8537
Tmean + Tbreadth + Mmean + Wintering + Numb. Gen. + Size + Range size	7	0.1953	0.1636	0.1298	2893.7631	2932.0042	2394028.1304	10441.4056	43.9271	0.8605
Tmean + Tbreadth + Mmean + Mbreadth + Wintering + Numb. Gen. + Size + Range size	8	0.1956	0.1604	0.1224	2895.6518	2937.3693	2403362.5154	10524.7554	44.2917	0.8673

b) Best Subsets Regression for Birds

Predictors	Predictors	Adj. R-Square	Pred. R-Square	R-Square	AIC	SBC	MSEP	FPE	HSP	APC
Wintering	1	0.0547	0.0321	-0.02	992.2917	1002.1553	427193.1068	5082.9126	59.1511	0.9898
Wintering + Numb. Gen.	2	0.0953	0.0626	-0.0024	990.4653	1002.7948	413737.2911	4978.1225	57.9781	0.9693
**Tbreadth + Wintering + Numb. Gen.**	**3**	**0.1487**	**0.1071**	**0.0335**	**987.1779**	**1001.9733**	**394089.2931**	**4794.4018**	**55.8983**	**0.9334**
Tmean + Tbreadth + Wintering + Numb. Gen.	4	0.1850	0.1347	0.0528	985.3835	1002.6449	381928.8460	4697.5206	54.8422	0.9144
Tmean + Tbreadth + Wintering + Numb. Gen. + Size	5	0.1933	0.1328	0.003	986.4963	1006.2235	382779.2717	4759.1540	55.6514	0.9262
Tmean + Tbreadth + Wintering + Numb. Gen. + Size + Range size	6	0.2026	0.1319	-0.0024	987.4856	1009.6787	383147.4798	4814.9550	56.4101	0.9370
Tmean + Tbreadth + Mmean + Wintering + Numb. Gen. + Size + Range size	7	0.2126	0.1319	-0.0047	988.3852	1013.0443	383182.6726	4866.6249	57.1385	0.9469
Tmean + Tbreadth + Mmean + Mbreadth + Wintering + Numb. Gen. + Size + Range size	8	0.2128	0.1208	-0.0164	990.3590	1017.4840	388042.0812	4980.2192	58.6146	0.9688

c) Best Subsets Regression for Butterflies

Predictors	Predictors	Adj. R-Square	Pred. R-Square	R-Square	AIC	SBC	MSEP	FPE	HSP	APC
**Mmean**	**1**	**0.1074**	**0.0912**	**0.0166**	**671.4709**	**677.6001**	**406705.1521**	**7385.3669**	**132.1299**	**0.9575**
Mmean + Wintering	2	0.1639	0.0996	-0.0441	673.7400	685.9983	388124.2902	7440.5308	133.3680	0.9289
Mmean + Wintering + Numb. Gen.	3	0.2123	0.1351	-0.0072	672.3449	686.6463	372713.7957	7266.8766	130.5838	0.9066
Tmean + Tbreadth + Mmean + Wintering	4	0.2270	0.1343	-0.0649	673.2695	689.6139	372919.4502	7392.9235	133.2690	0.9216
Tmean + Tbreadth + Mmean + Wintering + Numb. Gen.	5	0.2547	0.1482	-0.068	673.1933	691.5807	366771.7153	7391.2795	133.7470	0.9207
Tmean + Tbreadth + Mmean + Mbreadth + Wintering + Numb. Gen.	6	0.2721	0.1507	-0.0964	673.8476	694.2781	365524.4694	7486.1856	136.0691	0.9318
Tmean + Tbreadth + Mmean + Mbreadth + Wintering + Numb. Gen. + Size	7	0.2731	0.1339	-0.1345	675.7669	698.2405	372611.9740	7753.9476	141.6587	0.9643
Tmean + Tbreadth + Mmean + Mbreadth + Wintering + Numb. Gen. + Size + Range size	8	0.2731	0.1151	-0.1777	677.7666	702.2832	380537.8098	8044.3525	147.8169	0.9995

Table S6. Summary table full models and variable omission results. a) Main model (Same as Table 2 in main text), b) Alternative model 1 where we used the 0.75 quantile of distribution points to measure shift in northern range edge, c) Alternative model 2 where we used the cv (relative niche breadth) instead of sd (absolute niche breadth) to measure niche breadth, and d) Alternative model 3 where we used the GDD instead of MAT to describe the thermal niche and PREC instead of SWC to describe the moisture niche. In bold statistically significant effects (<0.05) of variables according to estimated t-values (to the left) and AIC values that indicate decreased model fit if variable is dropped (ΔAIC >2; to the right). Because of heteroscedasticity in residuals (Text S1), the alternative model 2 for butterflies was re-fitted with robust standard errors and the summary table presents the refitted model. The variable omission using *drop1* cannot be conducted for robust models, wherefor these results are based on the basic linear regression. Tmean = mean thermal niche; Tbreadth = breadth of thermal niche; Mmean = mean moisture niche; Mbreadth = breadth of moisture niche; Wintering = overwintering mode; Numb. Gen. = number of generations or broods per season; Size = body size; Range size = range size across Europe.

a) Main model (Same as Table 2 in main text)

	Summary table					Variable omission				
	Parameter	Estimate	Std. Error	t value	Pr(>|t|)	Variable dropped	ΔDf	ΔSum of Sq	RSS	AIC
Moth	(Intercept)	12.481	25.578	0.488	0.626	<none>			7730.0	842.86
	**Mean thermal niche**	35.881	6.163	5.822	**<0.001**	**Mean thermal niche**	1	1129.25	8859.3	**873.45**
	**Thermal niche breadth**	-31.571	5.604	-5.633	**<0.001**	**Thermal niche breadth**	1	1057.36	8787.4	**871.50**
	Wintering - Egg	23.241	28.324	0.821	0.413	**Wintering**	3	492.69	8222.7	**851.63**
	Wintering - Larvae	43.050	27.377	1.572	0.117					
	Wintering - Pupae	-6.433	27.409	-0.235	0.815					
	Parameter	Estimate	Std. Error	t value	Pr(>|t|)	Variable dropped	ΔDf	ΔSum of Sq	RSS	AIC
Bird	(Intercept)	-24.578	22.718	-1.082	0.2825	<none>			1383.5	250.68
	**Thermal niche breadth**	-23.890	7.686	-3.108	**<0.01**	**Mean thermal niche**	1	165.008	1548.5	**258.49**
	**Wintering - Resident**	44.574	17.360	2.568	**<0.05**	**Wintering**	2	127.370	1510.9	**254.34**
	Wintering - Short distance	6.054	18.577	0.326	0.7454	Number of broods	1	62.487	1446.0	252.53
	Number of broods >=2	30.919	16.165	1.913	0.0593					
	Parameter	Estimate	Std. Error	t value	Pr(>|t|)	Variable dropped	ΔDf	ΔSum of Sq	RSS	AIC
Butterfly	(Intercept)	32.743	9.691	3.379	0.00137	<none>			1359.9	184.81
	**Moisture niche breadth**	22.151	10.716	2.067	**<0.05**	**Moisture niche breadth**	1	109.63	1469.6	**187.23**

R^2= 0.253, 0.168, 0.075 and adjusted R^2 = 0.238, 0.128, 0.057 for moth, birds, and butterflies respectively

b) Alternative model 1 where we used the 0.75 quantile of distribution points to measure shift in northern range edge

	Summary table					Variable omission				
Moth	Parameter	Estimate	Std. Error	t value	Pr(>|t|)	Variable dropped	ΔDf	ΔSum of Sq	RSS	AIC
	(Intercept)	18.843	15.281	1.233	0.2188	<none>			2681.3	589.80
	**Mean thermal niche**	12.851	3.168	4.056	**<0.001**	**Mean thermal niche**	1	189.32	2870.6	**604.11**
	**Thermal niche breadth**	-17.595	3.114	-5.649	**<0.001**	**Thermal niche breadth**	1	367.28	3048.5	**618.48**
	Wintering - Egg	17.637	16.640	1.060	0.2903	**Wintering**	3	298.44	2979.7	**609.02**
	**Wintering - Larvae**	34.342	16.111	2.132	**<0.05**					
	Wintering - Pupae	-1.709	16.143	-0.106	0.9158					
Bird	Parameter	Estimate	Std. Error	t value	Pr(>|t|)	Variable dropped	ΔDf	ΔSum of Sq	RSS	AIC
	(Intercept)	34.935	5.639	6.195	<0.001	<none>			902.70	207.53
	Thermal niche breadth	-6.365	5.591	-1.138	0.258	Thermal niche breadth	1	13.765	916.47	206.85
Butterfly	Parameter	Estimate	Std. Error	t value	Pr(>|t|)	Variable dropped	ΔDf	ΔSum of Sq	RSS	AIC
	(Intercept)	24.293	7.248	3.352	<0.01	<none>			859.15	158.63
	**Mean thermal niche**	23.960	7.895	3.035	**<0.01**	**Mean thermal niche**	1	143.85	1003.00	**165.46**

R^2= 0.248, 0.015, 0.143 and adjusted R^2 = 0.232, 0.003, 0.128 for moth, birds, and butterflies respectively

c) Alternative model 2 where we used the cv (relative niche breadth) instead of sd (absolute niche breadth) to measure niche breadth

	Summary table					Variable omission				
	Parameter	Estimate	Std. Error	t value	Pr(>|t|)	Variable dropped	ΔDf	ΔSum of Sq	RSS	AIC
Moth	(Intercept)	12.479	25.583	0.488	0.626	<none>			7732.6	842.94
	**Mean thermal niche**	35.152	6.131	5.734	**<0.001**	**Mean thermal niche**	1	1095.7	8828.4	**872.61**
	**Thermal niche breadth**	-31.386	5.579	-5.625	**<0.001**	**Thermal niche breadth**	1	1054.8	8787.4	**871.50**
	Wintering - Egg	23.227	28.329	0.820	0.413	**Wintering**	3	493.2	8225.8	**851.72**
	Wintering - Larvae	43.060	27.382	1.573	0.117					
	Wintering - Pupae	-6.450	27.413	-0.235	0.814					
	Parameter	Estimate	Std. Error	t value	Pr(>|t|)	Variable dropped	ΔDf	ΔSum of Sq	RSS	AIC
Bird	(Intercept)	-24.402	22.649	-1.077	0.28450	<none>			1380.7	250.50
	**Thermal niche breadth**	-24.057	7.666	-3.138	**<0.01**	**Thermal niche breadth**	1	167.849	1548.5	**258.49**
	**Wintering -Resident**	44.758	17.348	2.580	**<0.05**	**Wintering**	2	128.246	1508.9	**254.23**
	Wintering - Short distance	6.128	18.556	0.330	0.74207	Number of broods	1	61.832	1442.5	252.32
	Number of broods - >=2	30.714	16.126	1.905	0.06038					
	Parameter	Estimate	Std. Error	t value	Pr(>|t|)	Variable dropped	ΔDf	ΔSum of Sq	RSS	AIC
Butterfly	(Intercept)	30.401	9.666	3.145	<0.001	<none>			1354.5	184.58
	**Moisture niche breadth**	23.548	11.098	2.122	**<0.05**	**Moisture niche breadth**	1	115.06	1469.6	**187.23**

R^2= 0.254, 0.170, 0.783 and adjusted R^2 = 0.238, 0.129, 0.061 for moth, birds, and butterflies respectively

d) Alternative model 3 where we used the GDD instead of MAT to describe the thermal niche and PREC instead of SWC to describe the moisture niche.

	Summary table					Variable omission				
Moth	Parameter	Estimate	Std. Error	t value	Pr(>|t|)	Variable dropped	ΔDf	ΔSum of Sq	RSS	AIC
	(Intercept)	15.597	25.612	0.609	0.543	<none>			7744.3	843.30
	**Mean thermal niche**	53.129	7.590	7.000	**<0.001**	Mean thermal niche	1	1635.59	9379.9	**887.10**
	**Thermal niche breadth**	-36.695	6.689	-5.486	**<0.001**	Thermal niche breadth	1	1004.50	8748.8	**870.45**
	Wintering - Egg	19.087	28.411	0.672	0.502	Wintering	3	650.15	8394.4	**856.57**
	Wintering - Larvae	43.701	27.378	1.596	0.112					
	Wintering - Pupae	-13.054	27.453	-0.475	0.635					
Bird	Parameter	Estimate	Std. Error	t value	Pr(>|t|)	Variable dropped	ΔDf	ΔSum of Sq	RSS	AIC
	(Intercept)	-20.694	23.766	-0.871	0.3865	<none>			1456.5	255.16
	**Thermal niche breadth**	-18.032	7.973	-2.262	**<0.05**	**Thermal niche breadth**	1	91.972	1548.5	**258.49**
	**Wintering -Resident**	42.404	17.926	2.365	**<0.05**	**Wintering**	2	117.927	1574.5	**257.93**
	Wintering - Short distance	3.970	19.163	0.207	0.8364	Number of broods	1	56.568	1513.1	256.47
	Number of broods - >=2	29.793	16.798	1.774	0.0799					
Butterfly	Parameter	Estimate	Std. Error	t value	Pr(>|t|)	Variable dropped	ΔDf	ΔSum of Sq	RSS	AIC
	(Intercept)	30.406	9.770	3.112	<0.01	<none>			1382.6	185.76
	Mean thermal niche	18.063	9.895	1.826	0.07355	Mean thermal niche	1	86.94	1469.6	187.23

R^2= 0.253, 0.125, 0.059 and adjusted R^2 = 0.237, 0.082, 0.041 for moth, birds, and butterflies respectively
